# Frameshift Variant in *AMPD2* in Cirneco dell’Etna Dogs with Retinopathy and Tremors

**DOI:** 10.3390/genes15020238

**Published:** 2024-02-13

**Authors:** Leonardo Murgiano, Jessica K. Niggel, Leontine Benedicenti, Matteo Cortellari, Arianna Bionda, Paola Crepaldi, Luigi Liotta, Geoffrey K. Aguirre, William A. Beltran, Gustavo D. Aguirre

**Affiliations:** 1 Division of Experimental Retinal Therapies, Department of Clinical Sciences & Advanced Medicine, University of Pennsylvania, Philadelphia, PA 19104, USA; jniggel@upenn.edu (J.K.N.); wbeltran@upenn.edu (W.A.B.); gda@upenn.edu (G.D.A.); 2 Sylvia M. Van Sloun Laboratory for Canine Genomic Analysis, University of Pennsylvania, Philadelphia, PA 19104, USA; 3 Matthew J. Ryan Veterinary Hospital, School of Veterinary Medicine, University of Pennsylvania, Philadelphia, PA 19104, USA; leoben@upenn.edu; 4 Department of Agricultural and Environmental Sciences—Production, Territory, Agroenergy, University of Milan, 20133 Milan, Italy; matteo.cortellari@unimi.it (M.C.); arianna.bionda@unimi.it (A.B.); paola.crepaldi@unimi.it (P.C.); 5 Department of Veterinary Sciences, University of Messina, 98168 Messina, Italy; luigi.liotta@unime.it; 6 Department of Neurology, Perelman School of Medicine, University of Pennsylvania, Philadelphia, PA 19104, USA; aguirreg@upenn.edu

**Keywords:** inherited canine disease, animal model, oculo-neurological syndrome, syndromic retinal condition

## Abstract

While the manifestations of many inherited retinal disorders are limited to loss of vision, others are part of a syndrome that affects multiple tissues, particularly the nervous system. Most syndromic retinal disorders are thought to be recessively inherited. Two dogs out of a litter of Cirneco dell′ Etna dogs, both males, showed signs of retinal degeneration, along with tremors and signs described as either atypical seizures or paroxysmal dyskinesias, while the other two male littermates were normal. We named this oculo-neurological syndrome CONS (Cirneco oculo-neurological syndrome), and undertook homozygosity mapping and whole-genome sequencing to determine its potential genetic etiology. Notably, we detected a 1-bp deletion in chromosome 6 that was predicted to cause a frameshift and premature stop codon within the canine *AMPD2* gene, which encodes adenosine monophosphate deaminase, an enzyme that converts adenosine 5′-monophosphate (AMP) to inosine 5’-monophosphate (IMP). Genotyping of the available Cirneco population suggested perfect segregation between cases and controls for the variant. Moreover, this variant was absent in canine genomic databases comprised of thousands of unaffected dogs. The *AMPD2* genetic variant we identified in dogs presents with retinal manifestations, adding to the spectrum of neurological manifestations associated with *AMPD2* variants in humans.

## 1. Introduction

Inherited retinal disorders arise in humans and other mammals due to variants in many different genes, each of which causes loss of sight due to either faulty development, metabolic dysfunction, or degeneration of the retinal photoreceptors [1]. As these disorders vary in their genetic etiology, inheritance can occur through autosomal recessive, dominant or X-linked mechanisms. Clinical manifestations also vary, depending in part on the type of cell impacted and time of onset. In human, retinitis pigmentosa [2] is the most common retinal disorder, with cone/cone–rod dystrophy [3] and Leber congenital amaurosis [4] also observed. While the manifestation of many of these retinal conditions is limited to loss of vision, some are part of a syndrome impacting other tissues, including the muscular, nervous, and cardiovascular systems, as well as the inner ear, skeleton, kidney, and liver [1]. More than 80 forms of syndromic retinal diseases associated with more than 200 genes have been described, with the majority being inherited recessively [1]. 

While syndromic retinal degenerations in humans have a diverse genetic etiology, many of the associated genes relate to failures in metabolism (which also include neurologic symptoms [5]) or ciliopathies [1] and can include congenital disorders of glycosylation [6], neuronal ceroid lipofuscinoses [7], mucopolysaccharidoses (MPSs) [8], and peroxisomal diseases [9]. Ciliopathies are caused by genetic variants in genes impacting the structure and function of the primary cilium, which is involved in many signaling pathways [10,11]. The outer segment of the photoreceptor is a highly modified primary sensory cilia, and therefore retinal diseases frequently co-manifest in ciliopathies [1,10,12], including Bardet–Biedl Syndrome [13], Joubert Syndrome [14], Usher Syndrome [15], Senior–Løken Syndrome [16], and Alström Syndrome [17]. Of note, most of these syndromes show great heterogeneity in phenotypic manifestations, with many patients exhibiting only some of the potential symptoms [1].

In addition to syndromic retinopathies that occur in metabolic diseases and ciliopathies, a link between structural and functional retinal degeneration and the development of neurodegenerative disorders in humans has also been noted [18]. As the retina develops from the invagination of an embryonic forebrain pouch, it can be considered an extension of the brain [1]. Indeed, the nervous system is frequently impacted in syndromic retinal disorders, including Alzheimer’s disease patients and animal models [19,20]. In addition, alterations in the structure of the retina and choroid, as well as in its micro-vasculature, occur as part of nonmotor symptoms observed in Parkinson’s Disease [21,22]. In Batten disease, the classic juvenile form of human neuronal ceroid lipofuscinosis, ocular symptoms manifest before neurologic symptoms [23], while in CLN8 neuronal ceroid lipofuscinosis, disease progression (onset of myoclonus and cognitive impairment) is rapid, and visual loss is often noted within two years of diagnosis [24]. In dogs, a frameshift mutation in canine TPP1 (the ortholog of the human CLN2-associated gene) is associated with neuronal ceroid lipofuscinosis in Dachshunds, which also presents with retinal pathology [25,26]. 

More than 300 genes and genetic loci have been associated with inherited retinal conditions (syndromic and not syndromic) in humans (RetNet: https://sph.uth.edu/RETNET/, accessed on 20 December 2023) and an increasing number of similar diseases are being detected in other mammals. In fact, large animal models of inherited human retinal degenerations have been identified in several mammalian species, including dogs, cats, sheep, horses and non-human primates [27]. As a result of common breeding practices, pure-bred dogs are a rich source of models for inherited disease (https://omia.org/home/, accessed on 20 December 2023). Identified variants in at least 40 genes have been associated with different forms of inherited retinal diseases in dogs. Of these, the majority are retinal degenerations and are collectively referred to as progressive retinal atrophy (PRA) in veterinary medicine [28] (ExpeRTs: https://www.vet.upenn.edu/experts, accessed on 20 December 2023). PRA can be diagnosed in virtually any dog breed. As such, investigation into the molecular etiology of retinal diseases in dogs is essential for the welfare and health of many breeds. Furthermore, by identifying molecular players and mechanisms driving retinal degenerations in these valuable large animal models, retinal therapies can be advanced to the clinical trial stage [29,30,31] and provide essential insight into the human disease [32,33,34]. 

The sighthound Cirneco dell’Etna (referred to as Cirneco, going forward) is a Mediterranean-type dog originally bred for hunting, and is one of the oldest Italian dog breeds, tracing back to ancient Sicily, Italy [35,36]. Currently, the distribution of this breed is very limited, and very few genetic studies involving Cirnecos have been carried out [36,37,38]. Italian dog population studies revealed that Cirnecos primarily cluster genetically with Ibizan Hound and Pharaoh Hound [38]. 

Four Cirnecos, all males and belonging to the same litter of eight, were brought to our attention. Of the four, two siblings presented with signs of retinal degeneration that was detected around ~4 years, along with paroxysmal neurological episodes consisting of head tremors and involuntary movements of one of the front limbs. The episodes were sporadic and would last only a few minutes and began at 2–2.5 years of age. The dogs were fully conscious during the episodes and would recover quickly. Their veterinarians described these episodes as either atypical seizures or movement disorders, such as paroxysmal dyskinesias. The other two available littermates were normal. We named this oculo-neurological syndrome CONS (Cirneco oculo-neurological syndrome) and, as we suspected a genetic etiology, we carried out mapping and whole genome sequencing to identify potential gene variants. 

## 2. Materials and Methods

### 2.1. Ethical Statement

The research and the examinations were conducted in full compliance with the Association for Research in Vision and Ophthalmology (ARVO) “Resolution on the Use of Animals in Ophthalmic and Vision Research”. The protocol was approved by the “Institutional Animal Care and Use Committee” (IACUCs), University of Pennsylvania (#806301).

### 2.2. Sample Collection and Phenotype Assessment

Blood- and buccal-swab-derived genomic DNA samples from a total of 35 Cirneco (two affected siblings and thirty-three unaffected, two of which were siblings of the affected, and one was their grandmother) were used for the study. Eleven of these dogs were from North America (NA) (including the four genotyped siblings), the rest from Europe (including the grandmother). DNA was extracted with the Illustra DNA extraction kit BACC2 (GE Healthcare), following manufacturer’s instructions. Genotyping was performed on 11 of these dogs (two cases, two unaffected siblings and seven unrelated healthy Cirnecos from NA) on a 220k Illumina canine SNP chip following standard protocols as recommended by the manufacturer. Pedigrees of all the genotyped dogs were retrieved from the Italian Kennel Club (ENCI) or The Breed Archive websites. The large pedigree was plotted with Kinship2 R package [39].

### 2.3. Mapping of the Causative Variant 

#### 2.3.1. Homozygosity Mapping

Homozygosity mapping was carried out with PLINK v.1.9 [40] to detect extended homozygosity intervals with shared alleles. In addition to the 11 dog SNP-chip dataset, we included 24 healthy Cirneco previously genotyped [36,38] as additional controls, for a total of 35. The 11-dog SNP dataset consisted of more than 220k evenly spaced SNPs, while the older dataset was genotyped on a 170k Illumina SNP chip. We used the commands --merge and --geno 0.1 to filter out a final count of 128,140. Homozygosity analysis was performed using the commands “--dog” (to account for the species-specific chromosome quantities), “--homozyg” and “--homozyg group”, and the standard parameters.

#### 2.3.2. Whole-Genome Sequencing

For both cases described, the PCR-free library was prepared as follows. Libraries of 300 bp insert size were prepared and Illumina NovaSeq6000 paired-end reads (2 × 100 bp) were collected. Fastq files were generated using Casava 1.8. A total of 1,521,125,559 reads (100 bp paired-end reads) were collected in total for the sequenced dogs (corresponding to an approximate average 32× and 45× coverage of the genome). The paired-end reads were mapped to the dog reference genome UU_Cfam_GSD_1.0 reference (Canfam4). The alignment was carried out using Burrows-Wheeler Aligner (BWA) version 0.5.9-r16 [41] under the default settings. The SAM file obtained by BWA was converted to a BAM file and sorted using samtools [42]. Duplicate marking was carried out using Picard tools (http://sourceforge.net/projects/picard/, accessed on 24 October 2023). Sorted BAM files were visualized using Integrative Genomics Viewer (IGV) [43].

#### 2.3.3. SNV and Short In-Del Discovery

Variant calling was carried out using GATK (version 2.4.9) [44], in the “HaplotypeCaller” mode, with the output set to variant call format (vcf, version 4.0); the raw calls for all samples and sites were flagged using the standard variant filtration module of GATK (shown in the “best practice” documentation of GATK, v.4). Prediction of the functional impact of the detected variants was carried out using SnpEff [45], comparing the data with the UU_Cfam_GSD_1.0 reference (Canfam4) assembly. Filtered candidate positions were converted to the Canfam 3.1 canine reference using the NCBI re-mapping service (https://www.ncbi.nlm.nih.gov/genome/tools/remap, accessed on 20 December 2023). In the affected cases, candidate variants mapped in the homozygous intervals were also filtered against the Dog Biomedical Variant Database Consortium (DBVDC), using the software BCFtools v1.4 [41,46]. Further searching was carried out in the European Variation Archive variant browser (https://www.ebi.ac.uk/eva/?Variant-Browser, accessed on 24 October 2023). 

#### 2.3.4. Structural Variant and Mobile Element Discovery

Delly2 [47] was used to detect five types of structural variants in the BAM files (Duplications, Inversions, Translocations, Insertions, and Deletions). BAM files from unrelated dogs belonging to other studies carried out either by our group or from publicly accessed databases were also used to filter out potential variants. The commands for deletions, insertions, inversion, translocations, and duplications were all executed separately. Each of these analyses was carried out focusing on the candidate region identified by mapping. Depth of coverage average for the whole genome and specific for the CFA6 (defined in a .bed file) interval were checked with samtools [42] depth and an awk line of code (samtools depth *bamfile* | awk ‘{sum+=$3} END { print “Average = “,sum/NR}’).

### 2.4. Genotyping

The *AMPD2* 1-bp deletion was verified in the cases and the available controls by re-sequencing targeted PCR products by Sanger sequencing. PCR primers were designed using PRIMER3 [48]. PCR products were run on 1.2% agarose gel, 0.5 μg/mL ethidium bromide. PCR products were amplified using flanking primers for the exon 15 deletion, F (5′- GCC TTA GAG TCC CAG AAC CA-3′ chr6: 42,698,090–42,698,109) and R (5′-ACC TGT ATT ACC TGG CCC AG-3′, chr6:42,698,279–42,698,298). Amplification was carried out with AmpliTaqGold360MasterMix (Life Technologies, Carlsbad, CA, USA). All the coordinates reported here are Canfam4. The wild-type amplicon spans 208 bp. Sequence data were visualized with 4Peaks (https://nucleobytes.com/4peaks/, accessed on 24 October 2023).

### 2.5. Population Structure

Genomic analyses were performed on a dataset containing 33 additional breeds of different origins as well as the 11 Cirneco subjects SNP-genotyped for this study (File S1). All the data used for the comparison were publicly available and genotyped with the Canine 170k SNP BeadChips [36,38,49].

After merging these data, the raw dataset, comprised of 463 individuals and 142,840 SNPs, underwent quality control using PLINK 1.9 [40], with the following thresholds: missing per individual 0.05, missing per genotype 0.05, and MAF 0.0001. Moreover, SNPs located on the sex chromosomes and directly related animals were excluded. The final dataset was composed of 437 dogs and 120,985 SNPs.

The genomic population structure of the included populations was explored with the following analyses: multidimensional scaling (MDS) analysis conducted with PLINK 1.9, bootstrapped Reynolds distances between breeds, bootstrapped identity-by-state (IBS) distances among single subjects using an in-house script, and ADMIXTURE [50]. The ADMIXTURE analysis was performed using a number of genetic clusters (K) ranging from 2 to 27; the best-fitting model was identified as the one with the lowest cross validation value. Phylogenetic tree representations were realized using PHYLIP.

## 3. Results

### 3.1. Phenotype Characterization 

**Figure 1:**
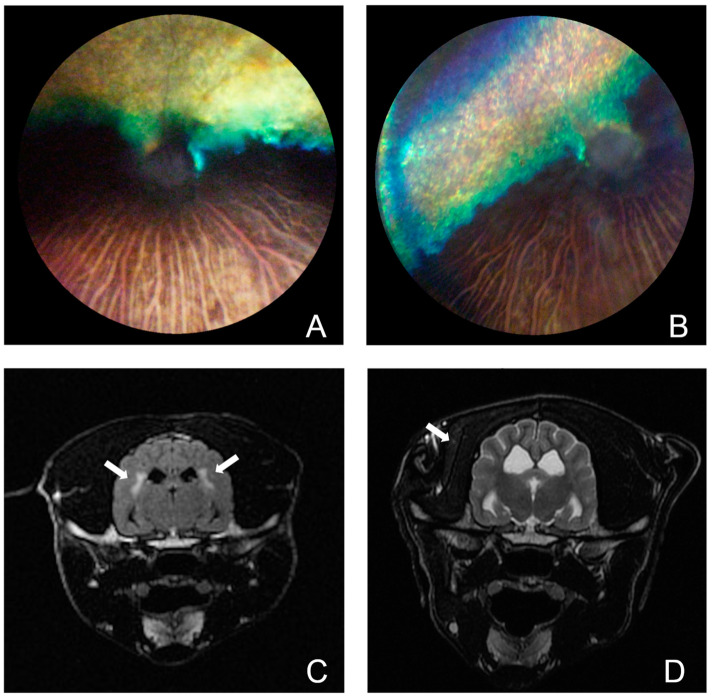
Retinal and cerebral phenotype. Left (**A**) and right (**B**) fundus pictures of a case (CRN4). Hyperreflectivity and marked attenuation/loss of retinal vessels along with pale atrophic optic discs indicate advanced stages of retinal degeneration. Sclerosis of the choroidal vessels is also present in the inferior non-tapetal region, accompanied by segmental choroid retinal atrophy. MRI of CRN4 (**C**) and CRN6 (**D**). (**C**) Transverse T2 FLAIR image at the level of the thalamus, where bilateral and symmetrical hyperintensities affecting the periventricular white matter tracts are depicted (arrows). (**D**): Transverse T2 weighted image at the level of the thalamus. In this case, no abnormalities were observed in the brain parenchyma. Moderate-to-severe left temporalis muscle atrophy is evident (arrow).

**Figure 2:**
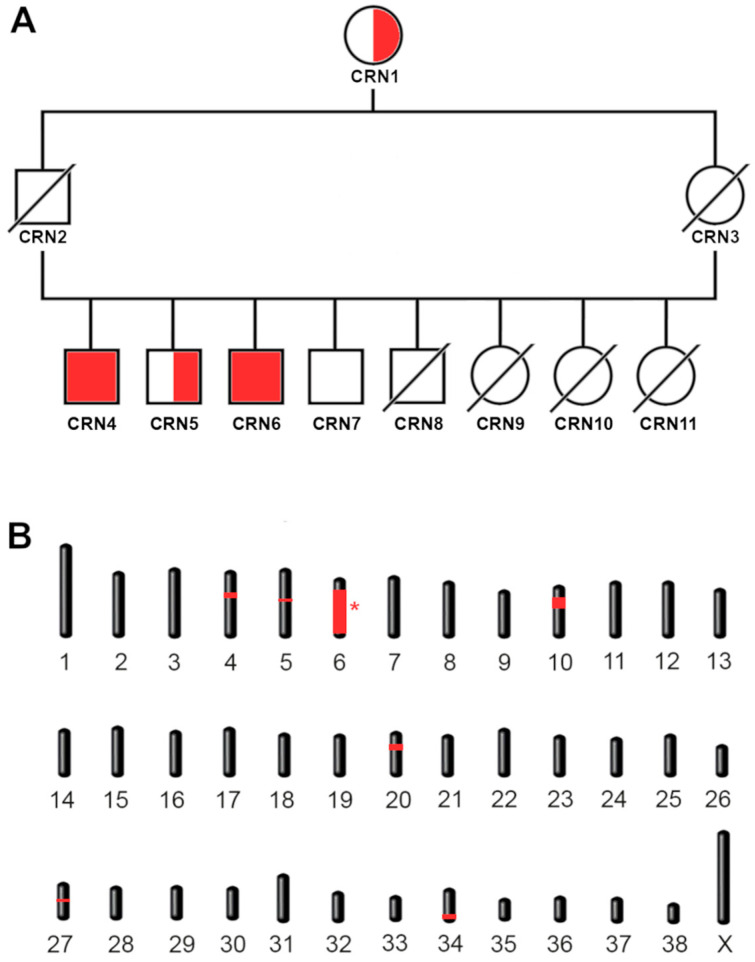
Family tree and mapping. (**A**) Family tree of the cases. The four siblings shown include the two cases. Homozygous cases CRN4 and CRN6 are shown with a red-filled shape. Carriers (CRN1 and CRN5) and wild-type (CRN7) controls are shown with a half-filled and empty shape, respectively. Males are indicated with a square, females with a circle. Missing samples that we were unable to genotype are indicated with a diagonal bar. The carrier grandmother CRN1 is shared by both parents. (**B**) Mapping of the candidate regions. Homozygous regions exclusive for the cases are shown in red. The interval containing the candidate variant is indicated with an asterisk.

Two Cirneco dogs were brought to our attention due to the deterioration of their vision and signs of retinal disease. Retinal abnormalities were first confirmed at ~4 years of age although, based on the advanced disease found at the time, the abnormalities were likely to be present much earlier (Figure 1A,B). Investigation into their clinical history revealed the presence of either some form of atypical seizures, manifesting as tremors without loss of consciousness, or paroxysmal dyskinesias, with an onset shortly after ~2–2.5 years of age. The cases were part of a litter of eight dogs, of which four, all males, were available for the study (Figure 2A). Blood samples from the cases and two normal littermates (deemed unaffected after eye exam of the fundus by a board-certified veterinary ophthalmologist) were collected. Blood samples from the parents of these dogs were unavailable due to distance and loss of contact with the owners, but they were reported as normal. Both dam and sire of the litter had the same mother, and she, too, was considered normal. 

Neurologic episodes in the first case (CRN4) started shortly after 2 years of age and consisted of head bobbing and vocalization, accompanied by lifting of the right front limb as if it were cramping. Eventually, the episodes started affecting the left front limb as well. The episodes would last 2–3 min and occurred in clusters over 2–3 days, but then did not reappear for several weeks. Neurological examination of CRN4 was mostly unremarkable. A full neurological examination was deemed difficult to perform due to the patient’s temperament (spinal reflexes were not tested). The dog had normal mentation and cranial nerve examinations, as well as a normal gait and postural reactions. The only abnormality detected reliably was cervical spinal pain. A preliminary diagnosis of either atypical seizure activity or paroxysmal dyskinesia was made and an MRI of the brain and cervical spinal cord, followed by cerebrospinal fluid (CSF) analysis, was performed. Analysis of the brain MRI by the authors (GKA, LB, clinical neurologists) showed a discrete area of bilaterally symmetric, peri-ventricular T2 hyperintensity (Figure 1C). This signal abnormality extended towards the internal capsule, where it was accompanied by increased T2 signal within the caudate nuclei bilaterally (right greater than left). There was also moderate ventriculomegaly involving the lateral ventricles. This appeared to result from diffuse thinning of cerebral white matter, most notable in the ventral temporal lobe. The results of the CSF analysis were within normal ranges (WBC count 1, reference range 0–5, protein content 17 mg/dL, reference range <30 mg/dL). The MRI of the cervical spine did not reveal any abnormalities. Complete blood cell count, biochemical profile and T4 testing did not reveal significant abnormalities, aside from an elevated ALKp at 710 IU/L (well above the reference range 5–131 IU/L). A video of CRN4 showing the head bobbing and gait alteration is shown in Supplementary Video S1.

The second case (CRN6) is reported to show early neurological signs by 2.5 years. CRN6 was examined by a neurologist at the age of 4 years due to concerns of visual deficits and acute onset of atrophy of the left temporalis muscle, with possible left facial nerve paralysis that was accompanied by sensitivity to touch and likely resulted from a traumatic accident (fall into a cellar). The neurological examination report indicated normal mentation, left-sided atrophy of the masticatory muscles, incomplete pupillary light reflexes and cervical pain. Gait analysis revealed an upper motor neuron gait affecting all four limbs, more pronounced in the thoracic limbs than in the pelvic ones. The dog had crossed extensor reflexes in all four limbs. A brain and cervical spine MRI were performed, which revealed chronic changes affecting the intervertebral disc at C6-7 with no significant compression of the spinal cord. The brain MRI analyzed by the authors (GKA, LB) showed ventriculomegaly similar in distribution but greater in severity as compared to CRN4; no focal T2 signal change was seen (Figure 1D). There was no parenchymal enhancement in the post-contrast images. A CSF analysis was not performed. Similar to CRN4, the owners reported that CRN6 began having seizures at the age of 2.5 years. Overall, a diffuse loss of cortical white matter volume, more prominent in the temporal lobes was observed in both cases. A summary of the clinical signs and their detection for both cases is reported in Table 1.

Around 4 years of age, both dogs showed clear signs of visual impairment. Examination of the fundus revealed thinning of the retinal vessels and an increased reflectivity of the tapetum. A diagnosis of an advanced stage of progressive retinal atrophy (PRA) was made (Figure 1A,B). 

### 3.2. Family Tree and Inheritance Mechanism

A total of two cases and two controls (four males) were initially examined. The two littermate cases showed both retinal and neurological signs, while the two controls did not. Information gathered about the immediate family of the cases revealed unaffected parents which shared the same unaffected mother but were unfortunately unavailable for sample collection. The available pedigree information, consisting of phenotypically unaffected parents with a shared unaffected grandmother, suggested that the most likely scenario was an autosomal recessive mode of inheritance. As the parents of the unaffected dogs were phenotypically normal, the chance of a fully penetrant autosomal dominant inheritance mechanism seemed unlikely, but we could not exclude X-linked recessive inheritance. Both cases had a panel of DNA tests for inherited retinal diseases in dogs (Embark™ dog DNA test) submitted by the owners, but they did not reveal any known marker for inherited diseases in dog.

### 3.3. Mapping of the Candidate Regions

As our limited pedigree analysis suggested the causative variant was inherited in an autosomal recessive manner, we opted for homozygosity mapping to identify the critical interval containing the causative variant. A batch of eleven Cirneco dogs, two cases, two unaffected siblings, and seven unrelated available unaffected controls, were genotyped using an Illumina Canine 220k SNP chip and merged with 24 Cirnecos previously genotyped on 170k Illumina canine SNP chips (see Section 2). Assuming a monogenic recessive inheritance pattern, we searched for extended regions of homozygosity, with the expectation that the disease allele and flanking chromosomal segments (most probably higher than 1 Mb, due to the sample pool size) in the affected dogs would be identical by descent (IBD). This initial filtering led us to consider the following shared regions: CFA4, CFA5, CFA6, CFA10, CFA20, CFA27 and CFA34 (Figure 2A). Once the critical homozygous candidate regions were confirmed, we converted their coordinates from Canfam 3.1 to the UU_Cfam_GSD_1.0 (Canfam4) reference, our reference of choice for the subsequent whole genome sequencing and variant calling, which includes sequences from the Dog10k database [51]. The entirety of the reported homozygous regions was counted as a candidate interval, as reported in Table 2. These were the only regions shared by all the cases but not by the controls. Results for homozygosity mapping are shown in Figure 2B, highlighting all the shared regions in red.

### 3.4. Variant Detection and Genotyping

Following selection of critical homozygous candidate regions, we next undertook whole genome sequencing, and reads were mapped against the UU_Cfam_GSD_1.0 reference (Canfam4). This reference was specifically selected, as it includes 1204 dogs from the Dog10k database (including six Cirnecos) as controls to effectively filter out previously known variants, since we predicted the causal variant to be novel. As an additional filtering measure, SNPs and small indels positions were converted to Canfam3.1 and filtered against the DBMVC database. This multi-step filtering eliminated a significant number of called SNP and small-indel variants in the interval as potential candidates. After filtering associated SNP or small-indel variants, we next assessed the presence of large deletion, insertions, inversions, duplications, and translocations. The variants were once again filtered against internal controls generated by our lab for other studies (47 dogs, no Cirnecos among them), and the UU_Cfam_GSD_1.0 (Canfam4) Dog10k database.

**Figure 3:**
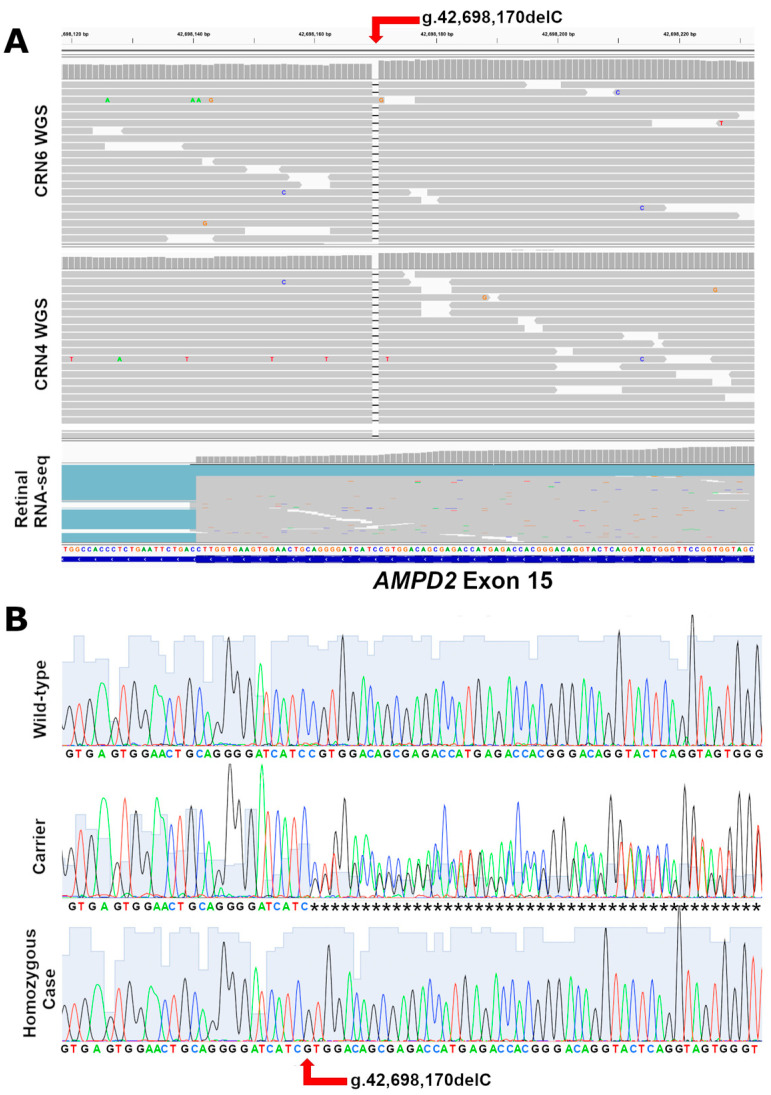
Sequencing of the putative causative variant. (**A**) Whole genome sequencing of two affected Cirneco (CRN4 and CRN6) and of unrelated retinal RNA-seq (confirming the exon and transcript in retina) shown in Integrate Genome Viewer. Note the 1-bp deletion (g.42,698,170delC). (**B**) Sanger sequencing of a control (top) a carrier (middle) and a case (bottom) showing the deletion of the coding C. Variant indicated with a red arrow.

Following this rigorous filtering process, 313 variants remained, of which 13 were large structural ones. Of the 313 variants, 312 were non-coding variants (File S2). The one coding variant identified in our sequenced Cirnecos and notably absent in all the control dogs sequenced in-house or present in the available databases resulted in a homozygous deletion in the coding region of the gene *AMPD2*, occurring at position CFA6: g.42,698,170delC, UU_Cfam_GSD_1.0, Canfam4 (Figure 3A). 

Due to the large size of the candidate region in chromosome 6, we next opted to exclude any de novo large deletions, possibly too large to be detected by our current tools, which could erroneously make the CFA6 critical interval appear homozygous, while actually being hemizygous. However, calculation of the average coverage of the whole genome of each case revealed the average chromosome for the CFA6 homozygous interval for CRN4 were, respectively, 45.25× (genome) and 45.92× (interval) and for CRN6, 32.02× (genome) and 32.99× (interval), excluding this possibility.

The *AMPD2* variant was compared with a re-annotated *AMPD2* transcript (predicted to have 18 exons in the retina, the same as the XM_038669870.1 canine *AMPD2* isoform in NCBI), revealing a 1-bp deletion in the 15th Exon of *AMPD2*, c.2,131delG (File S3A,B). The G deletion in the transcript results in a frameshift, leading to a terminally truncated protein p.D711Mfs12Stop (~50 AA missing out of the 826 AA of length) predicted to have lost 6.5% of its length (File S3C). As this variant resulted in a homozygous frameshift, we designed a PCR test based on two primers to sequence the DNA of any available Cirneco controls (Figure 3B, Table 3). This analysis revealed a perfect segregation in which the grandmother and a European Cirneco related to the cases were also found to be carriers. A larger family tree with the genotyped dogs was then built (File S4). Since the gene is expressed in the retina and previously associated with neurological conditions, we concluded that we had very likely identified the causative variant.

### 3.5. Variant Position and Gene Expression in Mammals

**Figure 4:**
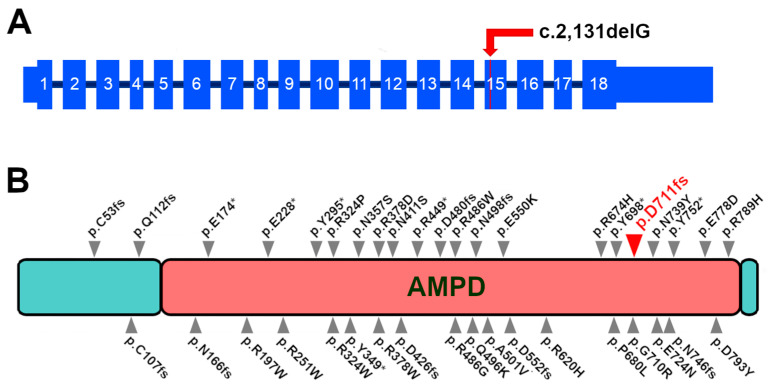
Position of the proposed causative variant. (**A**) Position of the c.2,131delG variant in the 15th exon of the canine *AMPD2* transcript. Exons shown as re-annotated from canine retinal RNA-seq. (**B**) Human AMPD2 protein with the position of the AMPD2 mutations associated with neurological diseases in Homo sapiens as reported in the literature [52,53,54,55,56,57]. The canine AMPD2 variant (position as realigned to the human protein) is reported in red and with the larger arrow. As for most pathological human variants, the mutation we report occurs within the AMPD domain.

The proposed causative genetic variant is positioned within the 15th exon of the *AMPD2* gene, near the 3′ of the transcript (Figure 4A). The predicted wild-type sequence of the canine AMPD2 protein was aligned with BLASTP against the human (taxid: 9606) database. The highest match result (97.34% identity, 100% query coverage) was with AMP deaminase 2 isoform 1 (NP_001355738.1). Subsequent alignment with ClustalOmega revealed that the predicted canine protein is 1 AA longer (826 AAs vs. the human 825). Additional alignment with comparable isoforms in other mammals revealed that the missing domain is highly conserved (File S3D,E). The mutation we report is predicted to fall within the wide AMPD domain of AMPD2 (Figure 4B).

While the re-annotation of the transcript was carried out with retinal RNA-seq generated in our laboratories, we also assessed the expression of *AMPD2*, *AMPD1*, and *AMPD3* transcripts in canine and human-retinal and -brain libraries. The complete list of samples is reported in File S2F. We detected *AMPD2* and to a lesser extent AMPD3 in the libraries, but no *AMPD1* in either species.

### 3.6. Population Genetics and Affected Family

Due to the small population of the breed and the low sample availability, and the partial European origin of the affected dogs, we next checked the population structure to determine (I) if Cirnecos within the American population were a suitable control pool and (II) if dogs directly related to the affected cases had signs of introgression from other breeds (to address the possibility that the variant did not originate in the Cirneco breed).

The population genetic structure analyses show that the two sampled cohorts of Cirnecos (i.e., the publicly available “CIRN” sampled in Italy and the “CRN” newly sampled for this study in the US) compared with 33 other breeds constitute a unique and homogeneous population. The breed list is reported in File S1.

**Figure 5:**
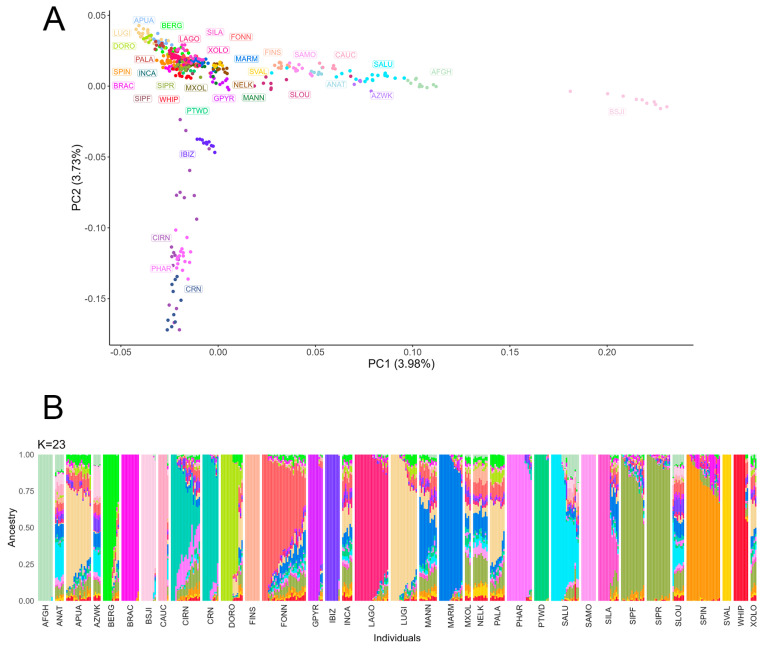
(**A**) Representation of the first two principal components (PCs) of the multidimensional scaling analysis of the individual identity-by-state distances. (**B**) Best-fitting model (number of clusters K = 23) obtained from the admixture analysis. Each bar represents a subject and each color a different cluster.

Indeed, despite a degree of dispersion observed in the MDS plot due to the different geographic origin of the samples (Figure 5A), both the phylogenetic tree based on Reynolds distances (File S5A) and on IBS (Figure S5B) clearly indicate a common genetic identity of the two sub-populations. This is further confirmed by the admixture analysis, where the two cohorts clustered as one population at the best-fitting K identified by the algorithm (K = 23) (Figure 5B).

## 4. Discussion

Through careful phenotyping, SNP genotyping, and whole genome sequencing, we were able to detect a 1-bp deletion in canine *AMPD2*, an adenosine monophosphate deaminase that converts AMP to IMP. This variant, which mapped to CFA6, caused a frameshift and premature stop codon within *AMPD2*, likely resulting in a terminally truncated protein. Genotyping of the available Cirneco population suggests perfect segregation of this variant among cases and controls for the condition. While we acknowledge the limitations of this study, mainly the low number of cases and of genotyped controls of this rare breed, we propose that the *AMPD2* variant most likely explains the phenotype of the newly identified oculo-neurological syndrome CONS. The alignment of the whole genome sequence of the two cases against UU_Cfam_GSD_1.0 (canfam4), along with available databases, allowed us to identify a likely causative variant, highlighting once more the importance of available genetic variant datasets for studies in animal models of retinal diseases, especially for less common breeds like Cirneco dell’Etna. It is currently unclear whether the genetic variant that we identified originated within the analyzed family (perhaps from the shared mother of the parents of the affected litter) or if it is more widespread in Europe or America. For the time being, we would recommend all Cirneco breeders test for the proposed variant, at the very least to monitor its frequency within the population. 

MRI findings of diffuse white matter loss and ventriculomegaly was present in both cases. Further, one case (CRN4) had focal white matter and a caudate signal change on T2-weighted imaging. While hypertension in this animal may have contributed to this finding, the bilateral caudate signal abnormality is also suggestive of metabolic injury, as is seen in toxic exposures (e.g., CO poisoning, heavy metals) or inherited genetic diseases (e.g., mitochondrial disorders, Neurofibromatosis type 1, and tubulinopathies) [58].

AMPD2 plays an important role in energy homeostasis [59], as it is involved in the metabolism of purine nucleotides, resulting in the generation of ATP and GTP which are critical for production and synthesis of nucleic acids [60]. De novo purine synthesis begins with the conversion of ribose-5-phosphate to IMP, which is then converted to ATP or GTP, based on cellular requirements [57]. AMPD functions as a homotetramer and participates in this pathway by converting AMP to IMP. While defects in purine nucleotide metabolism and synthesis have a significant impact on neurological disorders [57], extracellular ATP (and adenosine) are also potent signaling molecules within the retina [52]. An excess of extracellular ATP accelerates pathologic responses in retinal diseases, including age-related macular degeneration (AMD), glaucoma, and diabetic retinopathy [61,62]. Conversely, an accumulation of adenosine protects retinal cells against degeneration or inflammation [63]. 

Pontocerebellar hypoplasias (PCHs) are a group of rare inherited progressive neurodegenerative disorders. They can be classified into several subtypes that are associated with unique genes and variants, all of which share a prenatal onset and a characteristic time-dependent loss of the brain parenchyma [53,54,64]. In humans, PCH type 9 (PCH9) is associated with five different deleterious *AMPD2* homozygous variants (two premature terminations and three AA changes with an assumed recessive mechanism of inheritance) originally identified by Akizu and colleagues [57]. These patients showed microcephaly, delayed psychomotor development, and spasticity. Interestingly, six out of eight patients had seizures. Through brain imaging, the authors detected a ‘figure 8’ appearance of the brainstem in PCH9, as well as visible cerebral cortical atrophy and corpus callosum hypoplasia. The authors also reported that patient cells had increased levels of ATP and decreased levels of guanine nucleotides, suggesting a blockage of de novo purine biosynthesis that resulted in adenosine-mediated neurotoxicity and defective GTP-dependent protein translation [55,57]. 

Expanding the spectrum of phenotypes associated with *AMPD2*, Novarino and colleagues identified a homozygous frameshift mutation that occurred in affected members of a consanguineous family affected by spastic paraplegia [56]. The cases described were of a 20-year-old female and her 5-year-old nephew, both of whom walked unaided and with normal cognition. In addition, the MRI of the male individual did not reveal any abnormality in the cerebellum or brainstem, suggesting that even in humans the phenotypic variability is quite high. Finally, in eight new PCH9 patients and 17 previously reported individuals with biallelic *AMPD2* variants, Kortüm and colleagues [55] detected cases that lacked the “figure-8” midbrain shape, as well as a lack of visual contact, central visual impairment, a pale optic disc, or primary optic atrophy, again highlighting the phenotypic heterogeneity in human disease.

In the mouse, mutations of *AMPD2* are associated with hypercholesterolemia, resembling the human disorder [65]. Toyama and colleagues reported high proteinuria in an *AMPD2*-knockout mouse [66]. A comparison of the symptoms of human patients reveals that the phenotype of the affected Cirnecos does not entirely recapitulate human disease. While the canine cases present with retinal pathology, they lack the MRI abnormalities seen in the cerebellum and pons areas of human cases. However, almost all human patients demonstrate nonspecific white matter hyperintensities that closely resemble the lesions reported in one of the Cirneco cases. 

Due to its participation in ATP homeostasis, we speculate that the absence of a fully functional *AMPD2* in dogs explains the retinal phenotype found in Cirnecos. In a study of Nrl (Neural retina leucine zipper) gene mutant mice that show en masse conversion of rods into cones, Corbo and colleagues identified *Ampd2* within the top 63 genes most specifically expressed in cones within the ventral retina [67]. While *AMPD2*-related PCH in humans is not associated with retinal degeneration or primary optic atrophy, these manifestations are, in fact, found in other forms of PCH [64,68].

It must be noted that *AMPD2* is one of three AMP deaminases found in mammals. The tissue-specific expression patterns of *AMPD1* and *AMPD3* differ in humans and mice, which may explain divergencies in phenotype between human patients and *Ampd2*-null mice. Specifically, Akizu and colleagues [57] found that *Ampd2*-null mice have normal brain histology, while *Ampd2* and *Ampd3* double-knockout mice showed reduced body weight and brain size with little evidence of neuronal loss early in life. However, the double-knockout mice had a severely shortened life span (limited to 2 or 3 weeks) with “a neurodegenerative phenotype mostly affecting the CA3 pyramidal neurons in the hippocampus and some sparse pyknotic cells all over the cortex and the cerebellum” that was associated with behavioral gait disturbance observed after two weeks of age [57]. By analyzing the publicly available canine brain RNA-seq, we found that both *AMPD2* and *AMPD3* were expressed in the canine brain. Therefore, it is possible that the partial genetic redundancy for *Ampd2* and *Ampd3* observed in the mouse brain could also occur in dogs, leading to a less severe phenotype compared to the neurological syndromes detected in humans. This is, however, not the complete picture, because humans also seem to lack *AMPD1* in the RNA-seq libraries we analyzed.

The study has the obvious limitation of the low number of cases and controls, stemming from the rarity of their breed. Thus, further validation of the *AMPD2* variant that we identified as a putative causative mutation in CONS will require further investigation and a larger sample pool. Eventual confirmation of our study will expand the variable phenotype of *AMPD2*-related syndromes [56], as different mammalian genes in the same pathway exhibit unique or similar profiles in the context of genetic redundancies [69]. As such, this canine model may contribute to the understanding of *AMPD2*-related pathologies and serve as a model in which to test potential therapeutics for this disease, as well as for others in which purine nucleotide metabolism is impacted.

Our finding of a potential causative mutation based on two cases highlights how crucial it is for breeders/owners and veterinarians to report cases of observed pathologies to animal genetics researchers, particularly when a hereditary transmission mechanism is suspected. This reporting allows for the identification of the underlying cause of the pathology and the development of appropriate diagnostic tests. Based on our finding, it is advisable for Cirneco breeders to monitor their breeding lines and test their dogs for this mutation, especially if sporadic cases have been observed in their dogs. This proactive approach can help (I) confirm our proposed candidate variant as the causative mutation for CONS and (II) contain further spread of this mutation within the population (its frequency appears to be relatively low at present). Due to the small population size of the breed, and the early stage of discovery of the variant, it is up to the breeders to implement policies to avoid a genetic bottleneck. Indeed, the presence of the variant in dogs of European origin (and the overall closeness of those to the American pool tested) underscores the necessity of effective communication with breeders and the implementation of measures. Additionally, our findings suggest that this mutation originated from, and is currently limited to, a specific bloodline, emphasizing the importance of avoiding mating between closely related individuals. 

## 5. Conclusions

Through the use of whole genome sequencing, genotyping, and clinical examinations, we identified a variant in *AMPD2* as a likely causative mutation in a novel syndrome in a rare dog breed, the Cirneco dell’Etna. As such, we recommend that all Cirneco breeders test for the proposed variant. Additionally, our discovery in this canine model confirms and expands the variability of the phenotype spectrum occurring in *AMPD2* variants in human patients. 

## Figures and Tables

**Table 1:** Summary of the major ocular and neurological signs initially reported in both cases, and the age these were reported by the owners. Head bobbing and tremors began earlier (~2 years) in CRN4 compared to CRN6 (~2.5 years).

Manifestation	Detection
Ocular findings	
*Retinal degeneration*	~4 years
*Cataracts*	Unknown
Neurological findings	
*Head bobbing and tremors*	~2–2.5 years
*Atypical seizure activity*	~2.5 years
*Signs of cervical pain*	~2 years

**Table 2:** Candidate intervals obtained through homozygosity mapping of affected and unaffected siblings. Seven different candidate regions shared by the cases and not by the controls were identified. Note the large CFA6 interval. All coordinates are reported on the Canfam4 reference.

Chromosome	Start (bp)	End (bp)
chr4	30,472,934	33,342,556
chr5	41,535,525	42,456,236
chr6	13,160,052	78,103,814
chr10	18,760,527	30,002,859
chr20	16,053,617	20,940,104
chr27	5,115,516	6,885,858
chr34	32,724,176	39,490,788

**Table 3:** Distribution of the g. variant in our Cirneco sample pool and in a database of canine variants (multiple breeds, including six Cirneco). One of the three carriers marked with (*) is the shared grandmother of the cases (parents were not available). Additionally, one of the three carriers is a dog from the European pool of controls and closely related to the cases—see File S4.

Breed and Availability of Retinal Phenotype	N of Dogs	Genotype		
		wt/wt	wt/Del	Del/Del
**Cirneco Dell’etna—Cases**	2	0	0	2
**Cirneco Dell’etna—Unaffected**	33	30	3 *	0
**Dog10k**	1204	1204	0	0
**Total**	1239	1234	3	2

## Data Availability

SNP genotyping and whole-genome sequencing data have been made available at Dryad (https://datadryad.org/, accessed on 20 December 2023), link https://datadryad.org/stash/share/qtk7Gyp9I3J468hdiwPRkXdEI8zZqBU9rxb_w5m0Yec.

## Supplementary Materials

The following supporting information can be downloaded at: https://www.mdpi.com/article/10.3390/genes15020238/s1, Supplementary Video S1: CONS tremors manifestations, Supplementary File S1, Dataset used for the population structure and phylogenetic analyses; File S2, whole-genome variants list; File S3, Genetic variant and predicted impact; File S4, Extended family tree of the cases; File S5, Phylogenic trees based on Reynold distances and ISB.
