# Supplementary material for: Frameshift Variant in AMPD2 in Cirneco dell’Etna Dogs with Retinopathy and Tremors
Source: Genes (Basel). 2024 Feb 13;15(2):238. doi: 10.3390/genes15020238 (PMC10887799; doi:10.3390/genes15020238)
Supplement: Supplementary file 1 [file genes-15-00238-s001.zip › genes-2829890-supplementary/File_S1_Revision.docx]

**File S1** - Composition of the dataset used for the population structure and phylogenetic analyses.

| **Breed Code** | **Breed Name** | **Initial Dataset** | **Final Dataset** |
| --- | --- | --- | --- |
| AFGH | Afghan Hound | 10 | 10 |
| ANAT | Anatolian Shepherd | 6 | 6 |
| APUA | Pastore Apuano | 19 | 17 |
| AZWK | Azwahk Hound | 5 | 5 |
| BERG | Bergamasco Shepherd Dog | 15 | 11 |
| BRAC | Bracco Italiano | 12 | 12 |
| BSJI | Basenji | 10 | 10 |
| CAUC | Caucasian Shepherd Dog | 8 | 7 |
| CIRN | Cirneco dell'Etna (Italy) | 24 | 20 |
| CRN | Cirneco dell'Etna (USA) | 11 | 11 |
| DORO | Pastore d'Oropa | 15 | 15 |
| FINS | Finnish Spitz | 10 | 10 |
| FONN | Fonni's Dog | 30 | 30 |
| GPYR | Great Pyrenees | 10 | 10 |
| IBIZ | Ibizan Hound | 10 | 10 |
| INCA | Peruvian Inca Orchid | 7 | 7 |
| LAGO | Lagotto Romagnolo | 24 | 23 |
| LUGI | Lupino del Gigante | 23 | 18 |
| MANN | Mannara Dog | 12 | 12 |
| MARM | Maremma and the Abruzzi Sheepdog | 20 | 16 |
| MXOL | Miniature Xoloitzcuintle | 4 | 4 |
| NELK | Norwegian Elkhound | 10 | 10 |
| PALA | Pastore della Lessinia e del Lagorai | 10 | 10 |
| PHAR | Pharaoh Hound | 17 | 17 |
| PTWD | Portuguese Water Dog | 10 | 10 |
| SALU | Saluki | 19 | 19 |
| SAMO | Samoyed | 10 | 10 |
| SILA | Pastore della Sila | 14 | 14 |
| SIPF | Segugio Italiano Pelo Forte | 16 | 16 |
| SIPR | Segugio Italiano Pelo Raso | 16 | 16 |
| SLOU | Sloughi | 12 | 8 |
| SPIN | Spinone Italiano | 24 | 23 |
| SVAL | Swedish Valhund | 6 | 6 |
| WHIP | Whippet | 10 | 10 |
| XOLO | Xoloitzcuintle | 4 | 4 |
