# Supplementary material for: Frameshift Variant in AMPD2 in Cirneco dell’Etna Dogs with Retinopathy and Tremors
Source: Genes (Basel). 2024 Feb 13;15(2):238. doi: 10.3390/genes15020238 (PMC10887799; doi:10.3390/genes15020238)
Supplement: Supplementary file 1 [file genes-15-00238-s001.zip › genes-2829890-supplementary/File_S3_Revision.docx]

S3A – Re-annotated exons

>Exon_18

AGTTATTGAACGTGGTAGACTCTTGAGTGGGCTTTTTTTTTTCCTTCTTCCCATTTTTCTCTAGTCTCCAAATTTCTCCAAAGAAAAACTATTTTCTTTTCAGAGAAAACTATTATGGCCTTGTGGTTTTGAAATTATTTGCTCTCAGTAAATGCTTAAAAGAAAAATTTCAGAGAAACTAAAAATCCAGAAGCGTTTTTTAAAAAGATTGAGAGGTTGTGAAGAAACTCTTCAAATTGGGAACACTAGGCCTTAGCACACCCCGAGAACATACAGAGAACGTGGAATGGATGGTTGGGCACACGAGGCCCAGGCGGGGCTGGGGGCCCTGGACCAGAAGCTCATGGCCTCCGACCCACGGCCTGCCACTCAGACCTCCCGGGCCTGTTTCACCAACCAAGAACCAGCCAGGGTGGCACAATCTCCGGGTTAGCGTTCTGCTCTCGGGTTTGGTTTTAGTATCTATCCACGAGTGGAACGAATTAGTACGTGATGCCTTCTGGAGGCCGACGGACCCCTGGTCTTGGCCAGGAGTGAGCCTGGTGGCCAGCGGCCCTCGGGAGAACTGCCCAGAGGTGCCTGGTGAGACGCAGAGGCGCTGTgcccgggagaggcccgggagaggcccgggagaggccAGTGCACAGGAGGGGACGAAGCCTGCCTCCTGCTGCCTCACCAGGCCAATTCCTCACGCTCCTGCTGTTCCAGGGCTAACAGCTGGTGAAGCGCCAGCCCACCTACCTCCCCACTCAGCAGCTGCAGCAGCATCCACAGGCTTCTGTCACCAACCCCAGACCGCAGACAGCACAGGGCACATTCCACTCTTCTTCTTTACTCGGGCAACACACCCCAGCAACTTCTAGGCCCAAGGAGGTAGTCGGAGACCGTGCATCAAACACGAAGCGAAGCTAAGCTGCTTAAGCCTGGATTCCAAAGGCACAGGGTTGCTCCAACCCTCGTAAGAGAGCGTGCAGGTGGaacttgcgggtgtccacaccacaccttctgcctccgtcagaacctctgagaggaggacccggaaatggcatcttaagcaagttccccggaaaagcctgatgtcccaagtttgagaaccactgGCCTCAGGTCTGCTTGTGAGGGCAGACTCCCACGGTTACCCGAGGCTGGCAGTGAGGAAGCAGGAGGCGGAAGGAGATGCTAGCCGACACGTGGGTCTGACGGCCGTGTGCCCCAGACCGGGGGCCAAGTGTCCACTTCAGAGGGCCTGGGGAGCAGACGCATCCCCCGCGGGCTTCTCCGCCCTCCCAGCTTCCTGAGTGAGTCTCCTGCGTGCACACTCCTCAGCCAAGCCCCGTCACCCCCAGAGCGGCTCTGCCTCTGTCCACATCGCTCCTGGATCCACCCTCAGCCACCCCCACTGCCAATGGCTCCCTCTCTCCCGACAGAGATCCCACCTGCCTCATTACTTAAATAATCTTTATTTCTCATGCACTAGGAAAGATGGGATCGTATGAAACACTGCATTTCACAGCAAAGGCCTCTGACCAGAACACAACACAGGGGACCAGGTGGACTCAGGTCTGGTCAAAACTCGGGACCAGAGTGGGGATACCGCCCTCAGGGCTGCTGGCAGCTTCAGATGCCCCAGCTGCAGCGGCCCCGGACACAACACAGGGCAGCCGCCCCTCCCACAGAGGCACAGGGAGCTGACACCAGAGGAGCATGTGGACCAAAGAAGGGACAGGGGGGGCTGCTAGGCAGTGACTTGGTCAAGGCGGGGGTCTGGCACCTCCACGGCCCGAGTCGAGGGAGCCAGGGCTCTGCGCCCAGCTGTGCTCACCGCAGAGCCGCCGAAGGAGCTGTAGAGACCCCCCGGCCCGTCTGCTCCTCAGCCACATACACAAGACAACATCTGCCCAGGCGGGACCCCAAAGGCTAGAGGGGCCAGCCCCCCTCAACCATCCCTAGCCCCTGCAGACACACTGACGCCCACAGGACAGCCAGACTTCGGAGGTTCCTGGGACAGGGCCACCGGTGTGGCAGCCAGCAGACCGGAGGCCATCGAGTAGCCGAACCAAGACCAGGGCAGGGCAGATTCGGGGCACCATTTTCACTGAGGTGGCATGGgaacagaaacaggtcggagacgtgcaggacagaacagagtagaaaggacacatgcagacacaacagGGTGGGAGGGGCCGGAGGCTAAAACGTGGTCAAGGTGCCGAGAGGCAGGGGACACGGTGGGCCGGCCTCACTGAGGCCCCGGGCTGGTGGTGACCCCGGCCTCCTCTGGGATGGTCTCCAGCATCTCACTCTGGACAGCCTGTGTGATGAGCGCCAGCTCCTGGCACAGAGTCTCGTAGCGGTAGCCCACACGGATGTCCGGCACGTTGGTACGGCGGATGTCATTGCCCTCGGGGCCCTCCTTGGTATAGTTGGGTCCCAGCCAGTGGCTCTTCAC

>Exon_17

CTTGTGGGAGAAGCCGCTCATGAGCACACTGTTGCGTGCCAGCTCACACATGTCGCAGGAGCTGAGCTTCCACACCTGGGTGGCAATGCTATACTCCTCCATCAGTGGTTC

>Exon_16

CTTGGTGAAGTGGAACTGCAGGGGATCATCCGTGGACAGCGAGACCATGAGACCACGGGACAGGTACTCAGGTAGTGGGTTCCGGTGGTAGCTGAGGAAAAGGCTGTTGTTGCTGAGCGGAGACATGGCGATGCCAATCTGGGCCAGGTAATACAGGTACTGCAGGACCGGGGC

>Exon_15

CTTGCGCAGAAGCAGCCCATGAGAAATGTTCTCCGCCAGCATGAAGGCTGACACCAGGTGGTGGATGGGCCCGGCTTCCCCACAGTGCGGCCTCAGTACAAACGTGTGGAAGCCCCTCTGC

>Exon_14

CTGCGCAGGTGGTTCAGCATGGCCATGTTGGCAAAGGTGTAGTATAGGTAGTAGGCGTAGGGTGGGTTGTCCTCCTCCACCCAAGCCTCGGGGAGAGGGCTCTCCAGGTTGAAGACGTGATTCTCGGGCTTAGACTCATCATCCACGCTGTCAAAGCCATCCAC

>Exon_13

GTGTTCCAAGAAGAGGTGCAGCTCTGGGTGGCTGGCAGGGTGCACGGTGGCCTCAAACAGTGGCAGGAAGATGTTCTCCAGCATCTCCTGGAAGTTGGCCAGCTGGCCCTTGGTGCGGTACACGTCG

>Exon_12

AAGAGGCGGGGCACTTGCACGAGCCAGCGCACATTGGGAGAGTGTACTCGGTGCTTCACGGCCCAGCAGGCCAGCTTGTCCCACTCATCCCTAGAGCGCCCGTAGATGGAGAGCCGCAGCTCCGCATTCTGGTATTTGCTCTCCTCCAAATCTGACATCACCTC

>Exon_11

CTTGATAATGTGGGCAAAGTACTTTCCAGAAACCCTGTTGTCAGTCTTGATGAAGATCTCTCGGAGGACAGACTCCCCAATGGGGTTGTATTTGGCATTGAACTTGTCAAAACGATGGAAGGTGTTCCTGTC

>Exon_10

CGCGTGCATGTCCAGCGTGTCCACACTCAGGTCATAAGCAGTGAGATTCATGCTCTCGAACACCTCCCGCAACGTCTGCTCGCGGCCCTGCTCCACATGCACGATCTCCTCCAGGTGTCGCTTCATTGCCCGTTTGATGAAGCGCAGCAGGTGCTTCTGGTTCATGCAGGACGAGGCATGGATGTGTGTGTCCAC

>Exon_9

CTTCCGGATGTTGTAGAAATCTCGGTGTGGCACCTTCTTCTGGGCGGCCAGCTCCTTCATCTCGTTGAGCAGCACGTGCATCTGGAATTTGGAGCTCAGGTACTGTAGCCGGCGGTAGCAGAAGGACTTT

>Exon_8

ATAGGGCCATTGATTATCAGGGCCATCAACACATTGACATCTGCCACGAATTCCTGCAGGTCAGGGTACGGCAGCTCCACCTCTGAGCAA

>Exon_7

TGCTCATCGGCTTCCCTGCGGGTGTAGACATGCACCACGCCCTGCACCATGTGCAGACCCAAGCCCAGGTCCCCAGGCATGGTGCTTGGCTCACAGTACTCGTATGGGTGCTGCTCCAGCGCAGGGGGGTGCACCGGGGCAT

>Exon_6

CAGCGGACACAGGGGTGTCAGGGCCCTGCTCGTAGGTCCGCGTCTCCAGGGGCTTCTCAGCCAGCTGCTGCAGGTACCGGCGGGTCGTGGGGCAGAAGCTCTGCAGCGACAGGGCCATGTACTTCTCCCGGATGAAGAGCGCCCGCACCACACTCTTGGCTGCGTCCAGCAGGTCAGTGAACGGCAC

>Exon_5

CCCACACTTCTCCTCGCCAGAGATGGTGACCCGCTGAAATTCTCGCTCTAGCACCACGTCTCTTTCCCGTAGGCCCCGGTCGCCTTGCCCCTCACCCTTCTCCTTGTAGAGC

>Exon_4

TGGAGGTCTGAGTCACTGTCTGTCTTCAGGAAATCTTGCTTGGCCCGAAGCAGGATGTCTGGCTCCAGC

>Exon_3

TTGACATCCTGGCTGATCTGCCTCTCCAGGCGCTGCCTCCGCTCCTCCAGCTGCTCAATGGGGCTCTCCTCAGGGAACTCGTACGGGGCGCTGCGGAGCTCGCTCTCAGCCAGGGAGCGGCTGAACAGCTC

>Exon_2

CTCGGCGATCTCCTTGCATTTGCCATCCATTGACGTGCGTAGGTCGAGTGGGAAGTGCTTGAGGCAGGGGGCAGGGCCTGGCAGGGACCGGGCAGACTGCAGCGGAGGGGCCCCCAGCCCACCCCGAGCCT

>Exon_1

CTGGCACTGCAGAGGCGGCCTGCACGCTGGCCAGCTTCTTAAAGGGATATTTGGCCTTGGGGCTGCCGGGGCCGGGTGGGTTGGATGCCATGGCTCGCGCCGCGGGGACGGCGGGGGCACTCGGAGGCGCACGAGCGGGGCCCGGCCCTGCCACATCCAACCCCTTCCTCCGCCCCGCCCCCAGCGGGCCGACCGGTGCAGGAAGCAGAAGCCCCGCAGGCGGAAGAGGCCCTGGCCACGATTCCTGAGGTTGTCTAGGCAACCTCGGGAGGGGGCCTCCGTCGCC

S3B – Transcript reverse complement. Starting codon in green. Mutated base in red, bolded and underscored

>AMPD2

GGCGACGGAGGCCCCCTCCCGAGGTTGCCTAGACAACCTCAGGAATCGTGGCCAGGGCCTCTTCCGCCTGCGGGGCTTCTGCTTCCTGCACCGGTCGGCCCGCTGGGGGCGGGGCGGAGGAAGGGGTTGGATGTGGCAGGGCCGGGCCCCGCTCGTGCGCCTCCGAGTGCCCCCGCCGTCCCCGCGGCGCGAGCCATGGCATCCAACCCACCCGGCCCCGGCAGCCCCAAGGCCAAATATCCCTTTAAGAAGCTGGCCAGCGTGCAGGCCGCCTCTGCAGTGCCAGAGGCTCGGGGTGGGCTGGGGGCCCCTCCGCTGCAGTCTGCCCGGTCCCTGCCAGGCCCTGCCCCCTGCCTCAAGCACTTCCCACTCGACCTACGCACGTCAATGGATGGCAAATGCAAGGAGATCGCCGAGGAGCTGTTCAGCCGCTCCCTGGCTGAGAGCGAGCTCCGCAGCGCCCCGTACGAGTTCCCTGAGGAGAGCCCCATTGAGCAGCTGGAGGAGCGGAGGCAGCGCCTGGAGAGGCAGATCAGCCAGGATGTCAAGCTGGAGCCAGACATCCTGCTTCGGGCCAAGCAAGATTTCCTGAAGACAGACAGTGACTCAGACCTCCAGCTCTACAAGGAGAAGGGTGAGGGGCAAGGCGACCGGGGCCTACGGGAAAGAGACGTGGTGCTAGAGCGAGAATTTCAGCGGGTCACCATCTCTGGCGAGGAGAAGTGTGGGGTGCCGTTCACTGACCTGCTGGACGCAGCCAAGAGTGTGGTGCGGGCGCTCTTCATCCGGGAGAAGTACATGGCCCTGTCGCTGCAGAGCTTCTGCCCCACGACCCGCCGGTACCTGCAGCAGCTGGCTGAGAAGCCCCTGGAGACGCGGACCTACGAGCAGGGCCCTGACACCCCTGTGTCCGCTGATGCCCCGGTGCACCCCCCTGCGCTGGAGCAGCACCCATACGAGTACTGTGAGCCAAGCACCATGCCTGGGGACCTGGGCTTGGGTCTGCACATGGTGCAGGGCGTGGTGCATGTCTACACCCGCAGGGAAGCCGATGAGCATTGCTCAGAGGTGGAGCTGCCGTACCCTGACCTGCAGGAATTCGTGGCAGATGTCAATGTGTTGATGGCCCTGATAATCAATGGCCCTATAAAGTCCTTCTGCTACCGCCGGCTACAGTACCTGAGCTCCAAATTCCAGATGCACGTGCTGCTCAACGAGATGAAGGAGCTGGCCGCCCAGAAGAAGGTGCCACACCGAGATTTCTACAACATCCGGAAGGTGGACACACACATCCATGCCTCGTCCTGCATGAACCAGAAGCACCTGCTGCGCTTCATCAAACGGGCAATGAAGCGACACCTGGAGGAGATCGTGCATGTGGAGCAGGGCCGCGAGCAGACGTTGCGGGAGGTGTTCGAGAGCATGAATCTCACTGCTTATGACCTGAGTGTGGACACGCTGGACATGCACGCGGACAGGAACACCTTCCATCGTTTTGACAAGTTCAATGCCAAATACAACCCCATTGGGGAGTCTGTCCTCCGAGAGATCTTCATCAAGACTGACAACAGGGTTTCTGGAAAGTACTTTGCCCACATTATCAAGGAGGTGATGTCAGATTTGGAGGAGAGCAAATACCAGAATGCGGAGCTGCGGCTCTCCATCTACGGGCGCTCTAGGGATGAGTGGGACAAGCTGGCCTGCTGGGCCGTGAAGCACCGAGTACACTCTCCCAATGTGCGCTGGCTCGTGCAAGTGCCCCGCCTCTTCGACGTGTACCGCACCAAGGGCCAGCTGGCCAACTTCCAGGAGATGCTGGAGAACATCTTCCTGCCACTGTTTGAGGCCACCGTGCACCCTGCCAGCCACCCAGAGCTGCACCTCTTCTTGGAACACGTGGATGGCTTTGACAGCGTGGATGATGAGTCTAAGCCCGAGAATCACGTCTTCAACCTGGAGAGCCCTCTCCCCGAGGCTTGGGTGGAGGAGGACAACCCACCCTACGCCTACTACCTATACTACACCTTTGCCAACATGGCCATGCTGAACCACCTGCGCAGGCAGAGGGGCTTCCACACGTTTGTACTGAGGCCGCACTGTGGGGAAGCCGGGCCCATCCACCACCTGGTGTCAGCCTTCATGCTGGCGGAGAACATTTCTCATGGGCTGCTTCTGCGCAAGGCCCCGGTCCTGCAGTACCTGTATTACCTGGCCCAGATTGGCATCGCCATGTCTCCGCTCAGCAACAACAGCCTTTTCCTCAGCTACCACCGGAACCCACTACCTGAGTACCTGTCCCGTGGTCTCATGGTCTCGCTGTCCACG**G**ATGATCCCCTGCAGTTCCACTTCACCAAGGAACCACTGATGGAGGAGTATAGCATTGCCACCCAGGTGTGGAAGCTCAGCTCCTGCGACATGTGTGAGCTGGCACGCAACAGTGTGCTCATGAGCGGCTTCTCCCACAAGGTGAAGAGCCACTGGCTGGGACCCAACTATACCAAGGAGGGCCCCGAGGGCAATGACATCCGCCGTACCAACGTGCCGGACATCCGTGTGGGCTACCGCTACGAGACTCTGTGCCAGGAGCTGGCGCTCATCACACAGGCTGTCCAGAGTGAGATGCTGGAGACCATCCCAGAGGAGGCCGGGGTCACCACCAGCCCGGGGCCTCAGTGAGGCCGGCCCACCGTGTCCCCTGCCTCTCGGCACCTTGACCACGTTTTAGCCTCCGGCCCCTCCCACCctgttgtgtctgcatgtgtcctttctactctgttctgtcctgcacgtctccgacctgtttctgttcCCATGCCACCTCAGTGAAAATGGTGCCCCGAATCTGCCCTGCCCTGGTCTTGGTTCGGCTACTCGATGGCCTCCGGTCTGCTGGCTGCCACACCGGTGGCCCTGTCCCAGGAACCTCCGAAGTCTGGCTGTCCTGTGGGCGTCAGTGTGTCTGCAGGGGCTAGGGATGGTTGAGGGGGGCTGGCCCCTCTAGCCTTTGGGGTCCCGCCTGGGCAGATGTTGTCTTGTGTATGTGGCTGAGGAGCAGACGGGCCGGGGGGTCTCTACAGCTCCTTCGGCGGCTCTGCGGTGAGCACAGCTGGGCGCAGAGCCCTGGCTCCCTCGACTCGGGCCGTGGAGGTGCCAGACCCCCGCCTTGACCAAGTCACTGCCTAGCAGCCCCCCCTGTCCCTTCTTTGGTCCACATGCTCCTCTGGTGTCAGCTCCCTGTGCCTCTGTGGGAGGGGCGGCTGCCCTGTGTTGTGTCCGGGGCCGCTGCAGCTGGGGCATCTGAAGCTGCCAGCAGCCCTGAGGGCGGTATCCCCACTCTGGTCCCGAGTTTTGACCAGACCTGAGTCCACCTGGTCCCCTGTGTTGTGTTCTGGTCAGAGGCCTTTGCTGTGAAATGCAGTGTTTCATACGATCCCATCTTTCCTAGTGCATGAGAAATAAAGATTATTTAAGTAATGAGGCAGGTGGGATCTCTGTCGGGAGAGAGGGAGCCATTGGCAGTGGGGGTGGCTGAGGGTGGATCCAGGAGCGATGTGGACAGAGGCAGAGCCGCTCTGGGGGTGACGGGGCTTGGCTGAGGAGTGTGCACGCAGGAGACTCACTCAGGAAGCTGGGAGGGCGGAGAAGCCCGCGGGGGATGCGTCTGCTCCCCAGGCCCTCTGAAGTGGACACTTGGCCCCCGGTCTGGGGCACACGGCCGTCAGACCCACGTGTCGGCTAGCATCTCCTTCCGCCTCCTGCTTCCTCACTGCCAGCCTCGGGTAACCGTGGGAGTCTGCCCTCACAAGCAGACCTGAGGCcagtggttctcaaacttgggacatcaggcttttccggggaacttgcttaagatgccatttccgggtcctcctctcagaggttctgacggaggcagaaggtgtggtgtggacacccgcaagttCCACCTGCACGCTCTCTTACGAGGGTTGGAGCAACCCTGTGCCTTTGGAATCCAGGCTTAAGCAGCTTAGCTTCGCTTCGTGTTTGATGCACGGTCTCCGACTACCTCCTTGGGCCTAGAAGTTGCTGGGGTGTGTTGCCCGAGTAAAGAAGAAGAGTGGAATGTGCCCTGTGCTGTCTGCGGTCTGGGGTTGGTGACAGAAGCCTGTGGATGCTGCTGCAGCTGCTGAGTGGGGAGGTAGGTGGGCTGGCGCTTCACCAGCTGTTAGCCCTGGAACAGCAGGAGCGTGAGGAATTGGCCTGGTGAGGCAGCAGGAGGCAGGCTTCGTCCCCTCCTGTGCACTggcctctcccgggcctctcccgggcctctcccgggcACAGCGCCTCTGCGTCTCACCAGGCACCTCTGGGCAGTTCTCCCGAGGGCCGCTGGCCACCAGGCTCACTCCTGGCCAAGACCAGGGGTCCGTCGGCCTCCAGAAGGCATCACGTACTAATTCGTTCCACTCGTGGATAGATACTAAAACCAAACCCGAGAGCAGAACGCTAACCCGGAGATTGTGCCACCCTGGCTGGTTCTTGGTTGGTGAAACAGGCCCGGGAGGTCTGAGTGGCAGGCCGTGGGTCGGAGGCCATGAGCTTCTGGTCCAGGGCCCCCAGCCCCGCCTGGGCCTCGTGTGCCCAACCATCCATTCCACGTTCTCTGTATGTTCTCGGGGTGTGCTAAGGCCTAGTGTTCCCAATTTGAAGAGTTTCTTCACAACCTCTCAATCTTTTTAAAAAACGCTTCTGGATTTTTAGTTTCTCTGAAATTTTTCTTTTAAGCATTTACTGAGAGCAAATAATTTCAAAACCACAAGGCCATAATAGTTTTCTCTGAAAAGAAAATAGTTTTTCTTTGGAGAAATTTGGAGACTAGAGAAAAATGGGAAGAAGGAAAAAAAAAAGCCCACTCAAGAGTCTACCACGTTCAATAACT

S3C – Translated predicted protein

>AMPD2_WT_Translated

MASNPPGPGSPKAKYPFKKLASVQAASAVPEARGGLGAPPLQSARSLPGPAPCLKHFPLDLRTSMDGKCKEIAEELFSRSLAESELRSAPYEFPEESPIEQLEERRQRLERQISQDVKLEPDILLRAKQDFLKTDSDSDLQLYKEKGEGQGDRGLRERDVVLEREFQRVTISGEEKCGVPFTDLLDAAKSVVRALFIREKYMALSLQSFCPTTRRYLQQLAEKPLETRTYEQGPDTPVSADAPVHPPALEQHPYEYCEPSTMPGDLGLGLHMVQGVVHVYTRREADEHCSEVELPYPDLQEFVADVNVLMALIINGPIKSFCYRRLQYLSSKFQMHVLLNEMKELAAQKKVPHRDFYNIRKVDTHIHASSCMNQKHLLRFIKRAMKRHLEEIVHVEQGREQTLREVFESMNLTAYDLSVDTLDMHADRNTFHRFDKFNAKYNPIGESVLREIFIKTDNRVSGKYFAHIIKEVMSDLEESKYQNAELRLSIYGRSRDEWDKLACWAVKHRVHSPNVRWLVQVPRLFDVYRTKGQLANFQEMLENIFLPLFEATVHPASHPELHLFLEHVDGFDSVDDESKPENHVFNLESPLPEAWVEEDNPPYAYYLYYTFANMAMLNHLRRQRGFHTFVLRPHCGEAGPIHHLVSAFMLAENISHGLLLRKAPVLQYLYYLAQIGIAMSPLSNNSLFLSYHRNPLPEYLSRGLMVSLSTDDPLQFHFTKEPLMEEYSIATQVWKLSSCDMCELARNSVLMSGFSHKVKSHWLGPNYTKEGPEGNDIRRTNVPDIRVGYRYETLCQELALITQAVQSEMLETIPEEAGVTTSPGPQ

> AMPD2_Mutant_Translated

MASNPPGPGSPKAKYPFKKLASVQAASAVPEARGGLGAPPLQSARSLPGPAPCLKHFPLDLRTSMDGKCKEIAEELFSRSLAESELRSAPYEFPEESPIEQLEERRQRLERQISQDVKLEPDILLRAKQDFLKTDSDSDLQLYKEKGEGQGDRGLRERDVVLEREFQRVTISGEEKCGVPFTDLLDAAKSVVRALFIREKYMALSLQSFCPTTRRYLQQLAEKPLETRTYEQGPDTPVSADAPVHPPALEQHPYEYCEPSTMPGDLGLGLHMVQGVVHVYTRREADEHCSEVELPYPDLQEFVADVNVLMALIINGPIKSFCYRRLQYLSSKFQMHVLLNEMKELAAQKKVPHRDFYNIRKVDTHIHASSCMNQKHLLRFIKRAMKRHLEEIVHVEQGREQTLREVFESMNLTAYDLSVDTLDMHADRNTFHRFDKFNAKYNPIGESVLREIFIKTDNRVSGKYFAHIIKEVMSDLEESKYQNAELRLSIYGRSRDEWDKLACWAVKHRVHSPNVRWLVQVPRLFDVYRTKGQLANFQEMLENIFLPLFEATVHPASHPELHLFLEHVDGFDSVDDESKPENHVFNLESPLPEAWVEEDNPPYAYYLYYTFANMAMLNHLRRQRGFHTFVLRPHCGEAGPIHHLVSAFMLAENISHGLLLRKAPVLQYLYYLAQIGIAMSPLSNNSLFLSYHRNPLPEYLSRGLMVSLSTMIPCSSTSPRNH

S3D – Homo sapiens AMPD2 aligned with Translated predicted WT and mutant canine AMPD2

Homo              MASYPSGSGKPKAKYPFKKRASLQASTAAPEARGGLGAPPLQSARSLPGPAPCLKHFPLD 60

Dog               MASNPPGPGSPKAKYPFKKLASVQAASAVPEARGGLGAPPLQSARSLPGPAPCLKHFPLD 60

Mutated_Dog       MASNPPGPGSPKAKYPFKKLASVQAASAVPEARGGLGAPPLQSARSLPGPAPCLKHFPLD 60

                  *** * * *.********* **:**::*.*******************************

Homo              LRTSMDGKCKEIAEELFTRSLAESELRSAPYEFPEESPIEQLEERRQRLERQISQDVKLE 120

Dog               LRTSMDGKCKEIAEELFSRSLAESELRSAPYEFPEESPIEQLEERRQRLERQISQDVKLE 120

Mutated_Dog       LRTSMDGKCKEIAEELFSRSLAESELRSAPYEFPEESPIEQLEERRQRLERQISQDVKLE 120

                  *****************:******************************************

Homo              PDILLRAKQDFLKTDSDSDLQLYKEQGEGQGDRSLRERDV-LEREFQRVTISGEEKCGVP 179

Dog               PDILLRAKQDFLKTDSDSDLQLYKEKGEGQGDRGLRERDVVLEREFQRVTISGEEKCGVP 180

Mutated_Dog       PDILLRAKQDFLKTDSDSDLQLYKEKGEGQGDRGLRERDVVLEREFQRVTISGEEKCGVP 180

                  *************************:*******.****** *******************

Homo              FTDLLDAAKSVVRALFIREKYMALSLQSFCPTTRRYLQQLAEKPLETRTYEQGPDTPVSA 239

Dog               FTDLLDAAKSVVRALFIREKYMALSLQSFCPTTRRYLQQLAEKPLETRTYEQGPDTPVSA 240

Mutated_Dog       FTDLLDAAKSVVRALFIREKYMALSLQSFCPTTRRYLQQLAEKPLETRTYEQGPDTPVSA 240

                  ************************************************************

Homo              DAPVHPPALEQHPYEHCEPSTMPGDLGLGLRMVRGVVHVYTRREPDEHCSEVELPYPDLQ 299

Dog               DAPVHPPALEQHPYEYCEPSTMPGDLGLGLHMVQGVVHVYTRREADEHCSEVELPYPDLQ 300

Mutated_Dog       DAPVHPPALEQHPYEYCEPSTMPGDLGLGLHMVQGVVHVYTRREADEHCSEVELPYPDLQ 300

                  ***************:**************:**:********** ***************

Homo              EFVADVNVLMALIINGPIKSFCYRRLQYLSSKFQMHVLLNEMKELAAQKKVPHRDFYNIR 359

Dog               EFVADVNVLMALIINGPIKSFCYRRLQYLSSKFQMHVLLNEMKELAAQKKVPHRDFYNIR 360

Mutated_Dog       EFVADVNVLMALIINGPIKSFCYRRLQYLSSKFQMHVLLNEMKELAAQKKVPHRDFYNIR 360

                  ************************************************************

Homo              KVDTHIHASSCMNQKHLLRFIKRAMKRHLEEIVHVEQGREQTLREVFESMNLTAYDLSVD 419

Dog               KVDTHIHASSCMNQKHLLRFIKRAMKRHLEEIVHVEQGREQTLREVFESMNLTAYDLSVD 420

Mutated_Dog       KVDTHIHASSCMNQKHLLRFIKRAMKRHLEEIVHVEQGREQTLREVFESMNLTAYDLSVD 420

                  ************************************************************

Homo              TLDVHADRNTFHRFDKFNAKYNPIGESVLREIFIKTDNRVSGKYFAHIIKEVMSDLEESK 479

Dog               TLDMHADRNTFHRFDKFNAKYNPIGESVLREIFIKTDNRVSGKYFAHIIKEVMSDLEESK 480

Mutated_Dog       TLDMHADRNTFHRFDKFNAKYNPIGESVLREIFIKTDNRVSGKYFAHIIKEVMSDLEESK 480

                  ***:********************************************************

Homo              YQNAELRLSIYGRSRDEWDKLARWAVMHRVHSPNVRWLVQVPRLFDVYRTKGQLANFQEM 539

Dog               YQNAELRLSIYGRSRDEWDKLACWAVKHRVHSPNVRWLVQVPRLFDVYRTKGQLANFQEM 540

Mutated_Dog       YQNAELRLSIYGRSRDEWDKLACWAVKHRVHSPNVRWLVQVPRLFDVYRTKGQLANFQEM 540

                  ********************** *** *********************************

Homo              LENIFLPLFEATVHPASHPELHLFLEHVDGFDSVDDESKPENHVFNLESPLPEAWVEEDN 599

Dog               LENIFLPLFEATVHPASHPELHLFLEHVDGFDSVDDESKPENHVFNLESPLPEAWVEEDN 600

Mutated_Dog       LENIFLPLFEATVHPASHPELHLFLEHVDGFDSVDDESKPENHVFNLESPLPEAWVEEDN 600

                  ************************************************************

Homo              PPYAYYLYYTFANMAMLNHLRRQRGFHTFVLRPHCGEAGPIHHLVSAFMLAENISHGLLL 659

Dog               PPYAYYLYYTFANMAMLNHLRRQRGFHTFVLRPHCGEAGPIHHLVSAFMLAENISHGLLL 660

Mutated_Dog       PPYAYYLYYTFANMAMLNHLRRQRGFHTFVLRPHCGEAGPIHHLVSAFMLAENISHGLLL 660

                  ************************************************************

Homo              RKAPVLQYLYYLAQIGIAMSPLSNNSLFLSYHRNPLPEYLSRGLMVSLSTDDPLQFHFTK 719

Dog               RKAPVLQYLYYLAQIGIAMSPLSNNSLFLSYHRNPLPEYLSRGLMVSLSTDDPLQFHFTK 720

Mutated_Dog       RKAPVLQYLYYLAQIGIAMSPLSNNSLFLSYHRNPLPEYLSRGLMVSLSTMIPCSSTSPR 720

                  **************************************************  * .    :

Homo              EPLMEEYSIATQVWKLSSCDMCELARNSVLMSGFSHKVKSHWLGPNYTKEGPEGNDIRRT 779

Dog               EPLMEEYSIATQVWKLSSCDMCELARNSVLMSGFSHKVKSHWLGPNYTKEGPEGNDIRRT 780

Mutated_Dog       NH---------------------------------------------------------- 722

                  :

Homo              NVPDIRVGYRYETLCQELALITQAVQSEMLETIPEEAGITMSPGPQ 825

Dog               NVPDIRVGYRYETLCQELALITQAVQSEMLETIPEEAGVTTSPGPQ 826

Mutated_Dog       ---------------------------------------------- 722

S3E – WT and mutant canine AMPD2 aligned with other mammals. Observe the high level of conservation.

Canis_lupus_f           MASNPPGPGSPKAKYPFKKLASVQAASAVPEARGGLGAPPLQSARSLPGPAPCLKHFPLD 60

Canis_lupus_mutant      MASNPPGPGSPKAKYPFKKLASVQAASAVPEARGGLGAPPLQSARSLPGPAPCLKHFPLD 60

Homo_sapiens            MASYPSGSGKPKAKYPFKKRASLQASTAAPEARGGLGAPPLQSARSLPGPAPCLKHFPLD 60

Mus_musculus            MAS-YPGPGKSKAKYPFKKRAGLQASAAAPEARSGLGASPLQSARSLPGNAPCLKHFPLD 59

Rattus_Norvegicus       MAS-YPGPGKSKAKYPFKKRASLQASAAAPEARSGLGASPLQSARSLPGTAPCLKHFPLD 59

Bos_taurus              MASYSSGPSNPKAKYPFKKRASLQVSFAVPEARGGLGAPPLQSARSLPGPAPCLKHFPLD 60

                        ***   * .. ******** *.:*.: *.****.**** ********** **********

Canis_lupus_f           LRTSMDGKCKEIAEELFSRSLAESELRSAPYEFPEESPIEQLEERRQRLERQISQDVKLE 120

Canis_lupus_mutant      LRTSMDGKCKEIAEELFSRSLAESELRSAPYEFPEESPIEQLEERRQRLERQISQDVKLE 120

Homo_sapiens            LRTSMDGKCKEIAEELFTRSLAESELRSAPYEFPEESPIEQLEERRQRLERQISQDVKLE 120

Mus_musculus            LRTSMDGKCKEIAEELFSRSLAESELRSAPYEFPEESPIEQLEERRQRLERQISQDVKLE 119

Rattus_Norvegicus       LRTSMDGKCKEIAEELFSRSLAESELRSAPYEFPEESPIEQLEERRQRLERQISQDVKLE 119

Bos_taurus              LRTSMDGKCKEIAEELFSRSLAESELRSAPYEFPEESPIEQLEERRQRLERQISQDVKLE 120

                        *****************:******************************************

Canis_lupus_f           PDILLRAKQDFLKTDSDSDLQLYKEKGEGQGDRGLRERDVVLEREFQRVTISGEEKCGVP 180

Canis_lupus_mutant      PDILLRAKQDFLKTDSDSDLQLYKEKGEGQGDRGLRERDVVLEREFQRVTISGEEKCGVP 180

Homo_sapiens            PDILLRAKQDFLKTDSDSDLQLYKEQGEGQGDRSLRERDV-LEREFQRVTISGEEKCGVP 179

Mus_musculus            PDILLRAKQDFLKTDSDSDLQLYKEQGEGQGDRGLWERDVVLEREFQRVIISGEEKCGVP 179

Rattus_Norvegicus       PDILLRAKQDFLKTDSDSDLQLYKEQGEGQGDRGLWERDVVLEREFQRVIISGEEKCGVP 179

Bos_taurus              PDILLRAKQDFLKTDSDSDFQLYKEKGEGQGDRGLWERDAVLEREFQRVTISGEEKCGVP 180

                        *******************:*****:*******.* ***. ******** **********

Canis_lupus_f           FTDLLDAAKSVVRALFIREKYMALSLQSFCPTTRRYLQQLAEKPLETRTYEQGPDTPVSA 240

Canis_lupus_mutant      FTDLLDAAKSVVRALFIREKYMALSLQSFCPTTRRYLQQLAEKPLETRTYEQGPDTPVSA 240

Homo_sapiens            FTDLLDAAKSVVRALFIREKYMALSLQSFCPTTRRYLQQLAEKPLETRTYEQGPDTPVSA 239

Mus_musculus            FTDLLDAAKSVVRALFIREKYMALSLQSFCPTTRRYLQQLAEKPLETRTYEQSPDTPVSA 239

Rattus_Norvegicus       FTDLLDAAKSVVRALFIREKYMALSLQSFCPTTRRYLQQLAEKPLETRTYEQSPDTPVSA 239

Bos_taurus              FTDLLDAAKSVVRALFIREKYMALSLQSFCPTTRRYLQQLAEKPLETRTYEQGPDTPVSA 240

                        ****************************************************.*******

Canis_lupus_f           DAPVHPPALEQHPYEYCEPSTMPGDLGLGLHMVQGVVHVYTRREADEHCSEVELPYPDLQ 300

Canis_lupus_mutant      DAPVHPPALEQHPYEYCEPSTMPGDLGLGLHMVQGVVHVYTRREADEHCSEVELPYPDLQ 300

Homo_sapiens            DAPVHPPALEQHPYEHCEPSTMPGDLGLGLRMVRGVVHVYTRREPDEHCSEVELPYPDLQ 299

Mus_musculus            DAPVHPPALEQHPYEHCEPSAMPGDLGLGLRMVRGVVHVYTRRDPDEHCPEVELPYPDLQ 299

Rattus_Norvegicus       DAPVHPPALEQHPYEHCEPSTMPGDLGLGLRMVRGVVHVYTRRDPDEHCPEVELPYPDLQ 299

Bos_taurus              DAPVHPPVLEQHPYERCEPSTMPADLGLGLRMVRGVVHVYTRREPDEHCSEVELPYPDLQ 300

                        *******.******* ****:**.******:**:*********: **** **********

Canis_lupus_f           EFVADVNVLMALIINGPIKSFCYRRLQYLSSKFQMHVLLNEMKELAAQKKVPHRDFYNIR 360

Canis_lupus_mutant      EFVADVNVLMALIINGPIKSFCYRRLQYLSSKFQMHVLLNEMKELAAQKKVPHRDFYNIR 360

Homo_sapiens            EFVADVNVLMALIINGPIKSFCYRRLQYLSSKFQMHVLLNEMKELAAQKKVPHRDFYNIR 359

Mus_musculus            EFVADVNVLMALIINGPIKSFCYRRLQYLSSKFQMHVLLNEMKELAAQKKVPHRDFYNIR 359

Rattus_Norvegicus       EFVADVNVLMALIINGPIKSFCYRRLQYLSSKFQMHVLLNEMKELAAQKKVPHRDFYNIR 359

Bos_taurus              EFVADVNVLMALIINGPIKSFCYRRLQYLSSKFQMHVLLNEMKELAAQKKVPHRDFYNIR 360

                        ************************************************************

Canis_lupus_f           KVDTHIHASSCMNQKHLLRFIKRAMKRHLEEIVHVEQGREQTLREVFESMNLTAYDLSVD 420

Canis_lupus_mutant      KVDTHIHASSCMNQKHLLRFIKRAMKRHLEEIVHVEQGREQTLREVFESMNLTAYDLSVD 420

Homo_sapiens            KVDTHIHASSCMNQKHLLRFIKRAMKRHLEEIVHVEQGREQTLREVFESMNLTAYDLSVD 419

Mus_musculus            KVDTHIHASSCMNQKHLLRFIKRAMKRHLEEIVHVEQGREQTLREVFESMNLTAYDLSVD 419

Rattus_Norvegicus       KVDTHIHASSCMNQKHLLRFIKRAMKRHLEEIVHVEQGREQTLREVFESMNLTAYDLSVD 419

Bos_taurus              KVDTHIHASSCMNQKHLLRFIKRAMKRHLEEIVHVEQGREQTLREVFESMNLTAYDLSVD 420

                        ************************************************************

Canis_lupus_f           TLDMHADRNTFHRFDKFNAKYNPIGESVLREIFIKTDNRVSGKYFAHIIKEVMSDLEESK 480

Canis_lupus_mutant      TLDMHADRNTFHRFDKFNAKYNPIGESVLREIFIKTDNRVSGKYFAHIIKEVMSDLEESK 480

Homo_sapiens            TLDVHADRNTFHRFDKFNAKYNPIGESVLREIFIKTDNRVSGKYFAHIIKEVMSDLEESK 479

Mus_musculus            TLDVHADRNTFHRFDKFNAKYNPIGESVLREIFIKTDNKISGKYFAHIIKEVMADLEESK 479

Rattus_Norvegicus       TLDVHADRNTFHRFDKFNAKYNPIGESVLREIFIKTDNKISGKYFAHIIKEVMSDLEESK 479

Bos_taurus              TLDVHADRNTFHRFDKFNAKYNPIGESVLREIFIKTDNRVSGKYFAHIIKEVMSDLEESK 480

                        ***:**********************************::*************:******

Canis_lupus_f           YQNAELRLSIYGRSRDEWDKLACWAVKHRVHSPNVRWLVQVPRLFDVYRTKGQLANFQEM 540

Canis_lupus_mutant      YQNAELRLSIYGRSRDEWDKLACWAVKHRVHSPNVRWLVQVPRLFDVYRTKGQLANFQEM 540

Homo_sapiens            YQNAELRLSIYGRSRDEWDKLARWAVMHRVHSPNVRWLVQVPRLFDVYRTKGQLANFQEM 539

Mus_musculus            YQNAELRLSIYGRSRDEWDKLARWAVNHKVHSPNVRWLVQVPRLFDVYRTKGQLANFQEM 539

Rattus_Norvegicus       YQNAELRLSIYGRSRDEWDKLARWAVNHRVHSPNVRWLVQVPRLFDVYRTKGQLANFQEM 539

Bos_taurus              YQNAELRLSIYGRSRDEWDKLARWAVTHRVHSPNVRWLVQVPRLFDVYRTKGQLANFQEM 540

                        ********************** *** *:*******************************

Canis_lupus_f           LENIFLPLFEATVHPASHPELHLFLEHVDGFDSVDDESKPENHVFNLESPLPEAWVEEDN 600

Canis_lupus_mutant      LENIFLPLFEATVHPASHPELHLFLEHVDGFDSVDDESKPENHVFNLESPLPEAWVEEDN 600

Homo_sapiens            LENIFLPLFEATVHPASHPELHLFLEHVDGFDSVDDESKPENHVFNLESPLPEAWVEEDN 599

Mus_musculus            LENIFLPLFEATVHPASHPELHLFLEHVDGFDSVDDESKPENHVFNLESPLPEAWVEEDN 599

Rattus_Norvegicus       LENIFLPLFEATVHPASHPELHLFLEHVDGFDSVDDESKPENHVFNLESPLPEAWVEEDN 599

Bos_taurus              LENIFLPLFEATIHPASHPELHLFLEHVDGFDSVDDESKPENHVFNLESPLPEAWVEEDN 600

                        ************:***********************************************

Canis_lupus_f           PPYAYYLYYTFANMAMLNHLRRQRGFHTFVLRPHCGEAGPIHHLVSAFMLAENISHGLLL 660

Canis_lupus_mutant      PPYAYYLYYTFANMAMLNHLRRQRGFHTFVLRPHCGEAGPIHHLVSAFMLAENISHGLLL 660

Homo_sapiens            PPYAYYLYYTFANMAMLNHLRRQRGFHTFVLRPHCGEAGPIHHLVSAFMLAENISHGLLL 659

Mus_musculus            PPYAYYLYYTFANMAMLNHLRRQRGFHTFVLRPHCGEAGPIHHLVSAFMLAENISHGLLL 659

Rattus_Norvegicus       PPYAYYLYYTFANMAMLNHLRRQRGFHTFVLRPHCGEAGPIHHLVSAFMLAENISHGLLL 659

Bos_taurus              PPYAYYLYYTFANMAMLNHLRRQRGFHTFVLRPHCGEAGPIHHLVSAFMLAENISHGLLL 660

                        ************************************************************

Canis_lupus_f           RKAPVLQYLYYLAQIGIAMSPLSNNSLFLSYHRNPLPEYLSRGLMVSLSTDDPLQFHFTK 720

Canis_lupus_mutant      RKAPVLQYLYYLAQIGIAMSPLSNNSLFLSYHRNPLPEYLSRGLMVSLSTMIPCSSTSPR 720

Homo_sapiens            RKAPVLQYLYYLAQIGIAMSPLSNNSLFLSYHRNPLPEYLSRGLMVSLSTDDPLQFHFTK 719

Mus_musculus            RKAPVLQYLYYLAQIGIAMSPLSNNSLFLSYHRNPLPEYLSRGLMVSLSTDDPLQFHFTK 719

Rattus_Norvegicus       RKAPVLQYLYYLAQIGIAMSPLSNNSLFLSYHRNPLPEYLSRGLMVSLSTDDPLQFHFTK 719

Bos_taurus              RKAPVLQYLYYLAQVGIAMSPLSNNSLFLSYHRNPLPEYLSRGLMVSLSTDDPLQFHFTK 720

                        **************:***********************************  * .    :

Canis_lupus_f           EPLMEEYSIATQVWKLSSCDMCELARNSVLMSGFSHKVKSHWLGPNYTKEGPEGNDIRRT 780

Canis_lupus_mutant      NH---------------------------------------------------------- 722

Homo_sapiens            EPLMEEYSIATQVWKLSSCDMCELARNSVLMSGFSHKVKSHWLGPNYTKEGPEGNDIRRT 779

Mus_musculus            EPLMEEYSIATQVWKLSSCDMCELARNSVLMSGFSHKVKSHWLGPNYTKEGPEGNDIRRT 779

Rattus_Norvegicus       EPLMEEYSIATQVWKLSSCDMCELARNSVLMSGFSHKVKSHWLGPNYTKEGPEGNDIRRT 779

Bos_taurus              EPLMEEYSIATQVWKLSSCDMCELARNSVLMSGFSHKVKSHWLGPSYTKEGPEGNDIRRT 780

                        :

Canis_lupus_f           NVPDIRVGYRYETLCQELALITQAVQSEMLETIPEEAGVTTSPGPQ 826

Canis_lupus_mutant      ---------------------------------------------- 722

Homo_sapiens            NVPDIRVGYRYETLCQELALITQAVQSEMLETIPEEAGITMSPGPQ 825

Mus_musculus            NVPDIRVGYRYETLCQELALITQAVQSEMLETIPEEVGIVMSPGP- 824

Rattus_Norvegicus       NVPDIRVGYRYETLCQELALITQAVQSEMLETIPEEVGIVMSPGP- 824

Bos_taurus              NVPDIRVGYRHETLCQELALITQAVQSEMLETIPEEGGITMSPGPQ 826

S3F – Human and Canine RNA-seq, ENA database IDs.

Human brain

Human cortex: ERR3196922, ERR3196920

Frontal lobe: SRR363691, SRR363686

Hyppocampus: SRR363689, SRR363685

Caudate nucleus: SRR363698, SRR363701

Human Retina: ERR5236623, ERR5236651, ERR5236614, ERR5236616

Canine brain: ERR351173, ERR348232

Canine retina: PRJNA382537 (study).
