# Supplementary material for: Frameshift Variant in AMPD2 in Cirneco dell’Etna Dogs with Retinopathy and Tremors
Source: Genes (Basel). 2024 Feb 13;15(2):238. doi: 10.3390/genes15020238 (PMC10887799; doi:10.3390/genes15020238)
Supplement: Supplementary file 1 [file genes-15-00238-s001.zip › genes-2829890-supplementary/File_S4_Revision.docx]

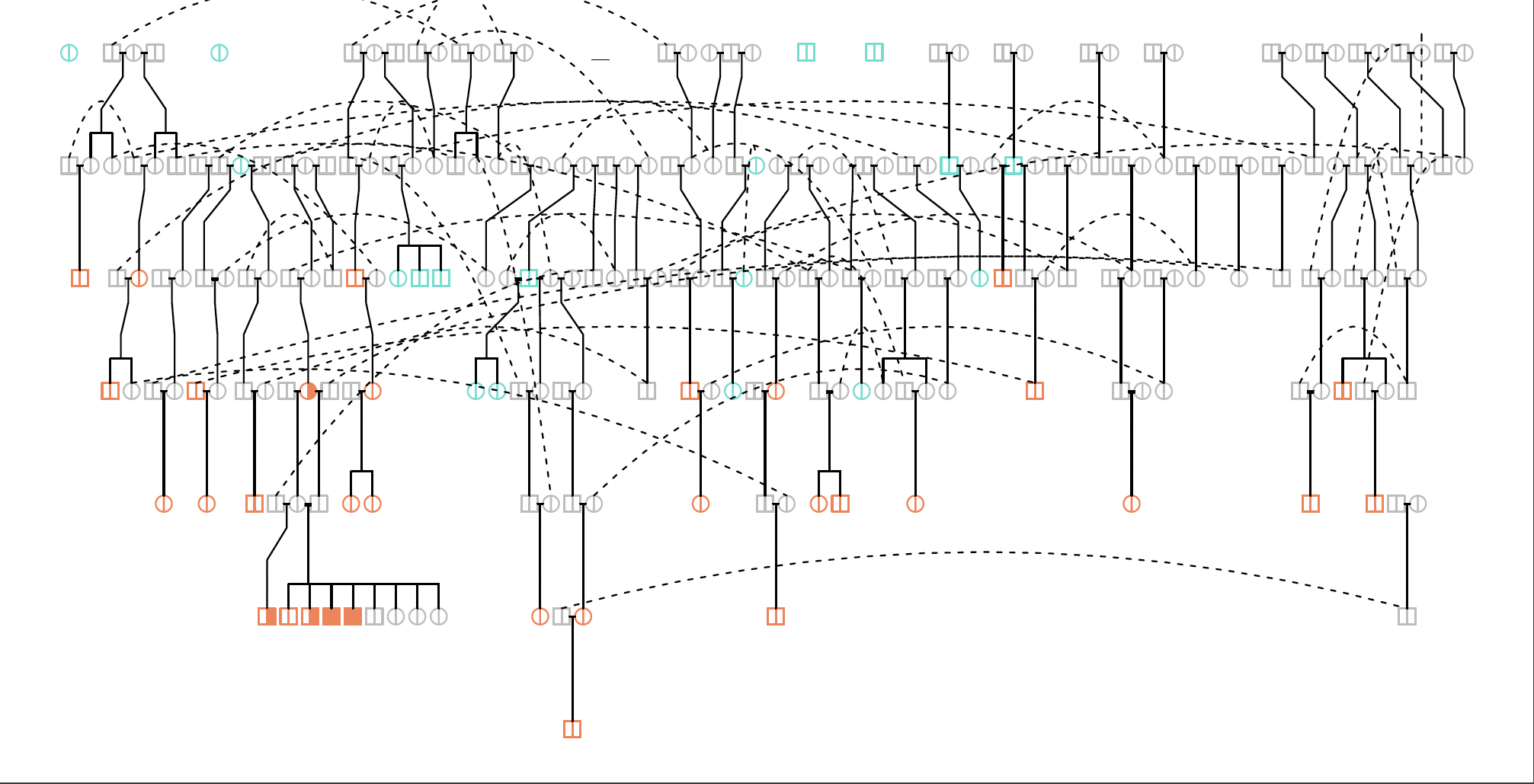


**Figure S4** - Pedigree representation of the genotyped Cirneco dell’Etna dogs. Circles represent female subjects and squares represent male subjects. Light blue color indicates that the dogs have been genotyped for comparison in the phylogenetic analyses, whereas individuals in orange color have been tested for the candidate variant. In this case, outlined symbols indicate homozygous wild dogs, half-colored symbols indicate heterozygous dogs, and symbols filled in color indicate dogs homozygous for the candidate variant.
