# Supplementary figures and images for: Frameshift Variant in AMPD2 in Cirneco dell’Etna Dogs with Retinopathy and Tremors
Source: Genes (Basel). 2024 Feb 13;15(2):238. doi: 10.3390/genes15020238 (PMC10887799; doi:10.3390/genes15020238)

A

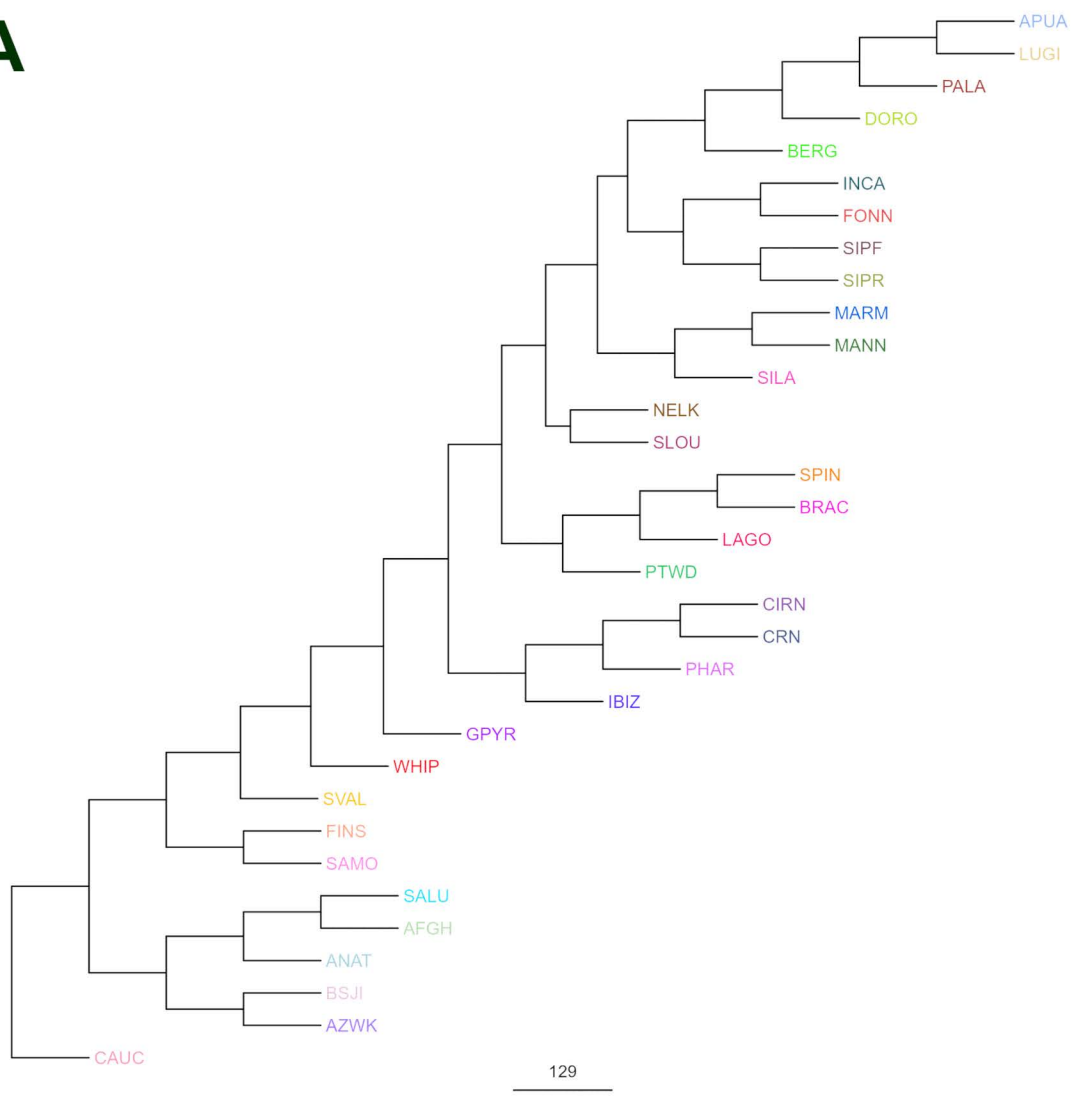

B

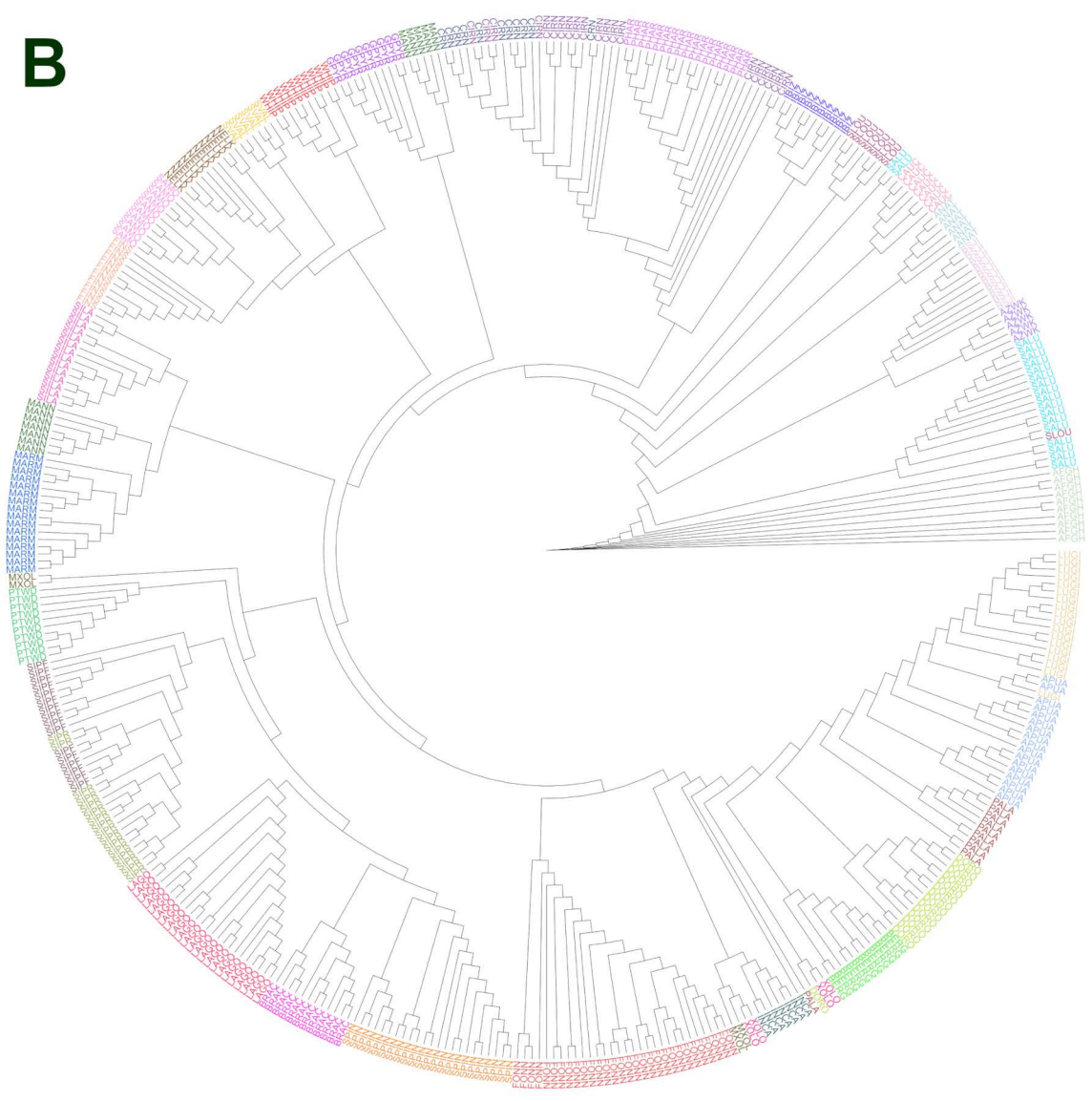

Supplement: Supplementary file 1 [file genes-15-00238-s001.zip › genes-2829890-supplementary/File_S5_Revision.pdf]
